# Efficacy of Glutamine in Treating Severe Acute Pancreatitis: A Systematic Review and Meta-Analysis

**DOI:** 10.3389/fnut.2022.865102

**Published:** 2022-06-14

**Authors:** Shi Dong, Zhenjie Zhao, Xin Li, Zhou Chen, Wenkai Jiang, Wence Zhou

**Affiliations:** ^1^The First School of Clinical Medicine, Lanzhou University, Lanzhou, China; ^2^Department of General Surgery, The First Hospital of Lanzhou University, Lanzhou, China; ^3^Department of General Surgery, Lanzhou University Second Hospital, Lanzhou, China

**Keywords:** glutamine, severe acute pancreatitis, treatment, prognosis, meta-analysis

## Abstract

**Objectives:**

The prognosis of severe acute pancreatitis (SAP) patients is closely related to early nutritional support. It is well-established that changes in glutamine (Gln), an important amino acid and nutritional supplement, can reflect disease severity. However, no consensus has been reached on the role of Gln nutrition therapy for SAP patients. We conducted this systematic review and meta-analysis to summarize and evaluate the advantages of Gln supplementation in SAP.

**Methods:**

PubMed, Web of Science, the Embase, Cochrane Library, and Chinese databases (CNKI, SinoMed, Wanfang, and VIP) were systematically searched for eligible studies that included glutamine supplementation in SAP patients from inception to October 31 2021, excluding non-SAP studies. Primary outcome measures included mortality, APACHE II score, complications, and length of hospital stay. The meta-analysis was registered with PROSPERO (CRD42021288371) and was conducted using Review Manager and Stata softwares.

**Results:**

This meta-analysis included 30 randomized controlled trials (RCTs) with a total of 1,201 patients. Six primary outcomes and six secondary outcomes were analyzed. For the primary outcomes, Gln supplementation was associated with lower mortality (OR = 0.38, 95% CI: 0.21–0.69, *P* = 0.001), total hospital stay (MD = −3.41, 95% CI: −4.93 to −1.88, *P* < 0.0001) and complications (OR = 0.45, 95% CI: 0.31–0.66, *P* < 0.0001) compared with conventional nutrition. Further subgroup analysis found that parenteral glutamine was more effective in reducing mortality. In terms of secondary outcomes, Gln supplementation helped restore liver, kidney and immune function, with significantly increased serum albumin (SMD = 1.02, 95% CI: 0.74–1.31, *P* < 0.00001) and IgG levels (MD = 1.24, 95% CI: 0.82–1.67, *P* < 0.00001), and decreased serum creatinine (Scr) (MD = −12.60, 95% CI: −21.97 to −3.24, *P* = 0.008), and inflammatory indicators such as C-reaction protein (CRP) (SMD = −1.67, 95% CI: −2.43 to −0.90, *P* < 0.0001).

**Conclusion:**

Although Gln supplementation is not routinely recommended, it is beneficial for SAP patients. Indeed, glutamine nutrition has little effect on some indicator outcomes but contributes to improving the prognosis of this patient population.

**Systematic Review Registration:** PROSPERO (york.ac.uk). Unique Identifier: CRD42021288371.

## Introduction

Acute pancreatitis (AP) is a predominant cause of acute abdomen during clinical practice and can be divided into mild, moderately severe, and severe AP according to its severity (1). Although most cases present with mild disease, more than 20% of patients progress to severe acute pancreatitis (SAP) (2, 3). SAP is characterized by hemorrhagic and necrotizing pancreatitis that frequently causes systemic complications and multiorgan failure, resulting in high mortality and poor prognosis (4, 5). As an important feature of SAP, high metabolism and increased protein decomposition rate can easily lead to malnutrition and subsequent immunosuppression, further aggravating SAP (6, 7). Early fasting is a crucial part of the conservative treatment of AP to reduce the secretion of pancreatic enzymes and the inflammatory response. However, it should be borne in mind that due to the high metabolic activity of the disease itself and the lack of exogenous nutritional supplements, early fasting can lead to the further progression of SAP, which ultimately increases the hospitalization time, medical costs and mortality (8, 9). Mounting evidence suggests that early enteral or parenteral nutrition support can help reduce multisystem organ failure (MOF), pancreatic infection complications and mortality, leading to a better prognosis (10).

As the most abundant amino acid in the human body, glutamine (Gln) is widely utilized by the liver, lung and intestine, and its supplementation may correct the negative nitrogen balance caused by SAP, reduce inflammation and improve prognosis (11, 12). However, much controversy surrounds the use of Gln in the treatment of SAP. Petrov et al. (13) found that Gln-supplemented enteral nutrition did not reduce total infectious complications relative to standard enteral nutrition (*P* = 0.53). This standpoint was confirmed in a meta-analysis by Jiang et al. (14), who demonstrated that Gln-containing enteral nutrition could significantly reduce the risk of multiple organ dysfunction syndromes (MODS) and death and shorten the length of hospital stay. Another study found that parenteral nutrition supplemented with Gln was more effective than enteral nutrition in reducing complications such as infection, pseudocyst and necrosis, but the difference was not statistically significant (15). This is significantly inconsistent with the study by Li et al., who believed that enteral nutrition was more meaningful than parenteral nutrition in reducing pancreatic infection and related complications in patients (OR, 0.41; 95% CI, 0.22–0.77; *P* = 0.006) (16). In a randomized controlled study that enrolled patients with SAP (*n* = 47), Gln-containing parenteral nutrition significantly increased serum albumin levels, decreased mortality and morbidity, shortened hospital stay, and improved patient nutritional status (17). The conclusion was consistent with Tina et al. (18). They confirmed that parenteral Gln supplementation significantly reduced the risk of infectious complications by enrolling 226 AP patients (RR = 0.59; 95% CI, 0.39–0.88; *P* ≤ 0.05) and mortality (RR = 0.26; 95% CI, 0.11–0.59; *P* ≤ 0.001) and shorter length of hospital stay (MD = −2.93 days; 95% CI, −4.70 to −1.15; *P* ≤ 0.001). However, the length of hospital stay was not consistent with Varsha et al. (MD = −1.35; 95% CI, −3.25 to 0.56, *P* = 0.17) (19). Therefore, much heterogeneity surrounds the application of Gln-containing nutritional support therapy in SAP. In addition, the 2019 Chinese Guidelines for the Diagnosis and Treatment of Acute Pancreatitis stated that the application of Gln preparations to SAP patients is beneficial to protect the intestinal mucosal barrier (20), but this was not mentioned in the American Gastroenterological Association Institute Guideline on Initial Management of Acute Pancreatitis (21). Although a meta-analysis by Li et al. (22) attempted to evaluate the efficacy of Gln-rich nutritional support for SAP patients, the small number of included studies limited robustness of the findings. To this end, we included updated studies and data for meta-analysis, including 30 randomized controlled trials (RCTs) and a large sample of more than 1,800 patients. In addition, we performed subgroup analyses of Gln supplementation patterns and even detailed supplementation doses, which were not addressed in previous studies. This will provide more reliable and robust evidence for the efficacy of Gln-containing nutritional therapy.

## Methods

Our study was registered in the PROSPERO database (CRD42021288371). This study was conducted according to the Cochrane Handbook 6.0, and the results were presented according to the PRISMA statement (23, 24).

### Search Strategy

Databases, including PubMed, Web of Science, the Embase, Cochrane Library, and Chinese databases (CNKI, SinoMed, Wanfang, and VIP), were searched until October 31, 2021. We used the following subject headings plus free words: “SAP (or severe acute pancreatitis, or acute necrotizing pancreatitis, or hemorrhagic necrotic pancreatitis)” and “Gln (or glutamine, or L-Glutamine, or D-Glutamine);” other quality conference papers and journals were also searched. The specific retrieval strategy for PubMed database is provided in Table 1.

**Table 1:** Retrieval strategy of PubMed.

**Number**	**Search strategy**
#1	“Glutamine” [MeSH]
#2	“L-Glutamine” [Title/Abstract]
#3	“L Glutamine” [Title/Abstract]
#4	“D-Glutamine” [Title/Abstract]
#5	“D Glutamine” [Title/Abstract]
#6	“Gln” [Title/Abstract]
#7	#1 OR #2 OR #3 OR #4 OR #5 OR #6
#8	“Pancreatitis, Acute Necrotizing” [MeSH]
#9	“Necrotizing Pancreatitis, Acute” [Title/Abstract]
#10	“Pancreatitis Necrotising” [Title/Abstract]
#11	“Acute Necrotizing Pancreatitis” [Title/Abstract]
#12	“Pancreatitis Necrotizing” [Title/Abstract]
#13	“Necrosis, Pancreatic” [Title/Abstract]
#14	“Hemorrhagic Necrotic Pancreatitis” [Title/Abstract]
#15	“Hemorrhagic Necrotic Pancreatitides” [Title/Abstract]
#16	“Necrotic Pancreatitis, Hemorrhagic” [Title/Abstract]
#17	“Pancreatitis, Hemorrhagic Necrotic” [Title/Abstract]
#18	“Severe Acute Pancreatitis” [Title/Abstract]
#19	“SAP” [Title/Abstract]
#20	#1 OR #2 OR #3 #8OR #9 OR #10 OR #11 OR #12 OR #13 OR #14 OR #15 OR #16 OR #17 OR #18 OR #19
#21	#7 AND #20

### Inclusion and Exclusion Criteria

Study selection was based on the following criteria: (1) study population: SAP patients; (2) intervention and comparison: treatment with Gln (supplemented enterally or parenterally) or without Gln for SAP patients; (3) outcomes: primary outcomes such as mortality, APACHE II score, shortening intensive care unit (ICU) hospital stay, total length of hospital stay, bloating recovery time, complications and secondary outcomes such as liver function indicators [serum albumin, alanine aminotransferase (ALT), aspartate aminotransferase (AST), and total bilirubin (TBIL)], kidney function indicators [serum creatinine (Scr) and blood urea nitrogen (BUN)], inflammatory indicators [C-reaction protein (CRP), interleukin-6 (IL-6), IL-8 and tumor necrosis factor α (TNF-α)], immune indicators (IgA and IgG) and serum amylase recovery time; and (4) study design: randomized controlled trials (RCTs).

Exclusion criteria: (1) research type: review, guideline, systematic review, animal experiment, cell experiment; (2) research content inconsistent with the theme and the full text unavailable; (3) duplicate studies.

### Definitions

The diagnosis of AP requires two of the following three criteria: (1) epigastric pain consistent with acute pancreatitis (acute, sudden, persistent and severe abdominal pain that can radiate to the back); (2) serum lipase activity; (3) Enhanced computed tomography (CT)/magnetic resonance imaging (MRI) showed typical AP imaging changes (pancreatic edema or peripancreatic effusion). Cases that met the dignostic criteria of AP with APACHE II score ≥ 8, Ranson score ≥ 3, CT grade D/E, accompanied by persistent (>48 h) organ dysfunction (especially shock, respiratory disorder, and renal insufficiency) and/or local complications (pancreatic necrosis, abscess, and pseudocyst) were diagnosed as SAP (1).

### Literature Screening and Data Extraction

Literature screening and data extraction were carried out by two researchers (Dong and Zhao), and any disagreements were resolved through third-party consultation (Li). A table was used to extract the relevant data of the included literature, including: (1) basic information of the study: first author, publication year, sample size, age, and gender ratio; (2) intervention measures: with or without Gln; (3) outcome indicators: mortality, APACHE II score, ICU hospital stay, total length of hospital stay, complications, bloating recovery time, liver function indicators, kidney function indicators, inflammatory indicators, immune indicators, and serum amylase recovery time.

### Literature Quality Assessment

The quality of each included study was assessed by the two investigators using tools in the Cochrane Handbook for the Systematic Review of Interventions (23). The evaluation content was divided into six aspects: (1) whether the random allocation method is correct; (2) whether the concealment of the allocation scheme is correct; (3) whether blinding is used; (4) whether the data results are complete; (5) whether there is selective reporting of research results; (6) whether there are other sources of bias.

### Statistical Analysis

All studies were analyzed using Review Manager version 5.3 and Stata 12SE. The Odd Ratio (OR) was used as the effect index for dichotomous variables, and the mean difference (MD) or standard mean difference (SMD) for continuous variables. Its estimates and 95% confidence interval (CI) were provided. The chi-square (χ^2^) and *I*^2^ tests were used to evaluate the heterogeneity. If significant heterogeneity was present among studies (*P* < 0.10 and *I*^2^ > 50%), a random-effects model was used; otherwise, a fixed-effects model was used (25). Sensitivity analysis was used to analyze sources of heterogeneity. A *P*-value <0.05 was statistically significant. Potential publication bias was assessed with funnel plots and Egger's test (26).

## Results

### Literature Search Results

**Figure 1:**
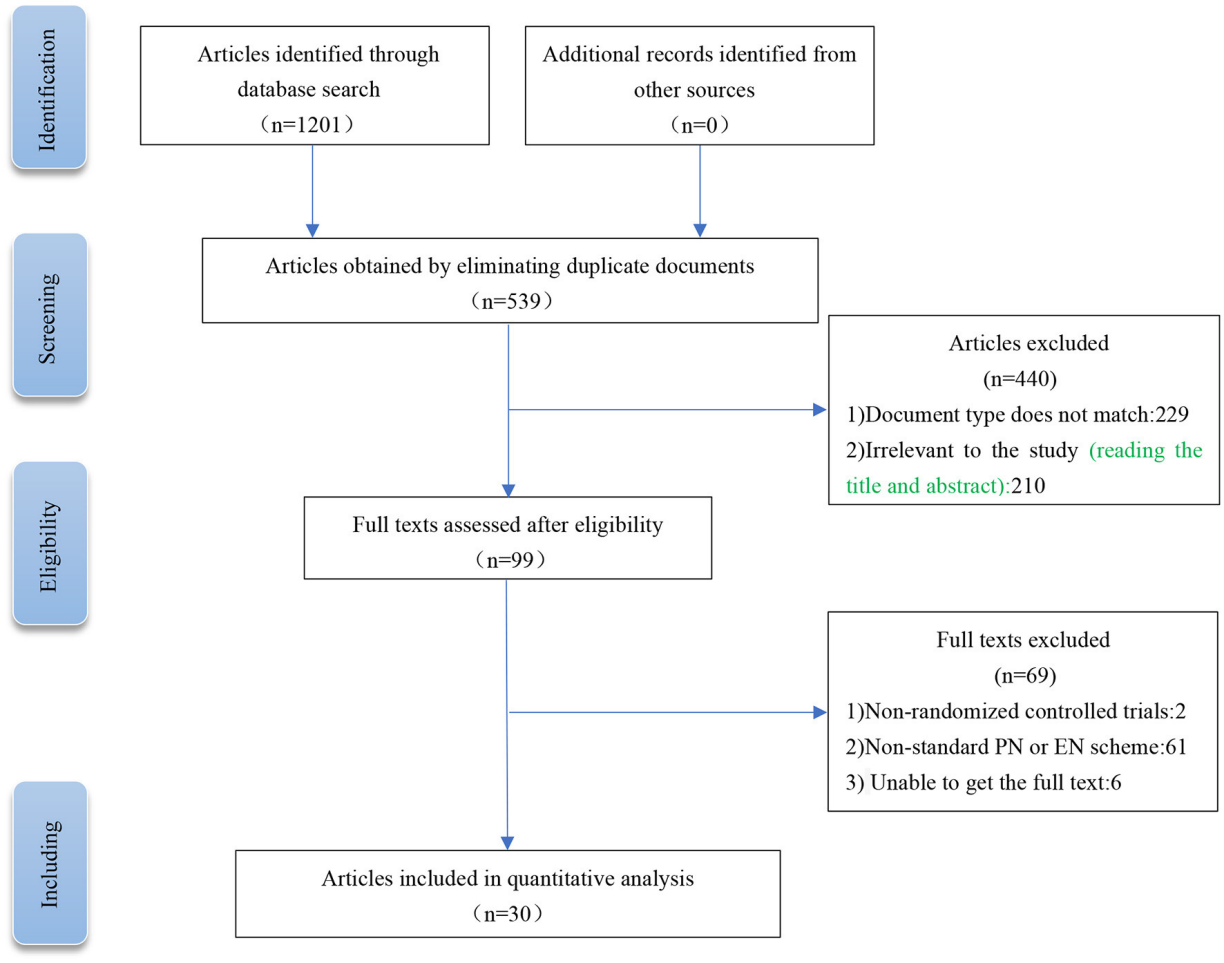
Current meta-analysis article searching and screening strategies.

A total of 1,201 related literatures were initially retrieved from the above databases. After screening, browsing the titles and abstracts, and reading the full texts, we finally included 30 RCTs (6, 12, 17, 27–53). The experimental group (nutritional support containing Gln) included 915 patients, and the control group (conventional nutritional support) included 917 patients. The PRISMA flow chart for literature screening is shown in Figure 1. The basic characteristics of the included studies are shown in Table 2.

**Table 2:** Summary of the basic characteristics of the included studies.

**Reference**	**Interventions**	**No. of subjects enrolled**	**Age (years, mean ± SD)**	**Sex (M/F)**	**APACHE II score**	**Outcomes**
He et al. (6)	TPN+Gln	20	39.4 ± 8.6	11/9	Unstates	A, D, E, F, G, K
	TPN	21	40.2 ± 7.8	11/10	Unstates	
Guo et al. (34)	TPN+Gln	20	Unstates	12/8	Unstates	A, G
	TPN	21	Unstates	14/7	Unstates	
Ding et al. (29)	TPN+Gln	10	Unstates	Unstates	Unstates	G, J
	TPN	10	Unstates	Unstates	Unstates	
Fuentes-Orozco (12)	TPN+Gln	22	43.8 ± 14.4	12/10	10.3 ± 1.6	A, C, D, G, I, J
	TPN	22	41.5 ± 14.2	12/10	10.7 ± 1.9	
Gu et al. (32)	TPN+Gln	30	Unstates	Unstates	Unstates	G
	TPN	30	Unstates	Unstates	Unstates	
Tong et al. (41)	EEN+Gln	16	41.5 ± 10.8	10/6	12.1 ± 3.7	B, I, J
	EEN	16	41.9 ± 11.4	9/7	12.1 ± 3.7	
Wang et al. (44)	TPN+Gln	23	Unstates	Unstates	Unstates	A, D, E, F, G, K
	TPN	25	Unstates	Unstates	Unstates	
Huang et al. (35)	TPN+Gln	24	63.6 ± 6.2	18/6	20.8 ± 3.5	B, G, I, J
	TPN	24	62.4 ± 4.8	16/8	21.0 ± 3.2	
Wu et al. (45)	EEN+Gln	15	37.24 ± 3.56	9/6	Unstates	A, D, F, G
	EEN	15	38.61 ± 2.51	10/5	Unstates	
Yang et al. (47)	EEN+Gln	14	42.82 ± 9.02	7/7	9.14 ± 0.77	B, C, D, I, J
	EEN	14	43.25 ± 8.73	7/7	9.21 ± 0.89	
Ran et al. (38)	TPN+Gln	25	54.3 ± 16.2	18/7	9.9 ± 2.4	B, C, D, G, H, I, J
	TPN	25	56.2 ± 15.9	20/5	9.5 ± 2.3	
Jin et al. (36)	EEN+Gln	26	Unstates	Unstates	12.5 ± 4.1	A, B, D, F
	EEN	23	Unstates	Unstates	12.0 ± 5.0	
Singh et al. (52)	EEN+Gln	41	40.78 ± 15.5	24/27	8.7 ± 4.4	A, F
	EEN	39	35.64 ± 13.0	25/14	7.1 ± 2.5	
Wa et al. (42)	EEN+Gln	54	59.3 ± 3.9	28/26	10.4 ± 1.2	B, G, H, I
	EEN	54	62.4 ± 4.1	31/23	10.5 ± 1.2	
Liu et al. (17)	TPN+Gln	24	40 ± 3.96	15/9	Unstates	A, C, D, F
	TPN	23	39.13 ± 4.46	14/9	Unstates	
Lei et al. (37)	EEN+Gln	38	Unstates	Unstates	16.5 ± 1.7	A, B, D, F, I, J, L
	EEN	38	Unstates	Unstates	15.9 ± 2.2	
Yin et al. (49)	TPN+Gln	20	41 ± 3.2	Unstates	Unstates	A, D, E, F, G, K
	TPN	20	41 ± 3.2	Unstates	Unstates	
Wang et al. (43)	EEN+Gln	49	51.85 ± 3.49	29/20	9.59 ± 1.33	G, I
	EEN	49	51.83 ± 3.52	28/21	9.63 ± 1.31	
Yang et al. (46)	EEN+Gln	34	Unstates	22/12	11.98 ± 1.42	I
	EEN	34	Unstates	21/13	12.08 ± 1.36	
Zhao et al. (51)	EEN+Gln	48	58.6 ± 3.8	Unstates	10.6 ± 1.2	B, G, I
	EEN	48	58.6 ± 3.8	Unstates	10.4 ± 1.2	
Cui et al. (28)	EEN+Gln	47	52.7 ± 8.3	32/15	9.7 ± 2.5	G, I, J
	EEN	47	53.5 ± 8.8	34/13	9.8 ± 2.7	
Gao et al. (31)	EEN+Gln	45	47.93 ± 6.24	24/21	Unstates	B, G, I
	EEN	45	48.56 ± 6.37	25/20	Unstates	
Yuan et al. (50)	EEN+Gln	23	51.32 ± 11.65	Unstates	Unstates	I, J
	EEN	24	51.32 ± 11.65	Unstates	Unstates	
Arutla et al. (53)	EEN+Gln	18	38.11 ± 16.3	17/11	8.6 ± 4.5	A, C, D, I
	EEN	22	39.77 ± 15.1	20/2	8.76 ± 3.7	
Ren et al. (39)	EEN+Gln	30	47.95 ± 7.79	21/9	11.34 ± 2.37	A, B, E, F, I, K
	EEN	30	51.71 ± 7.09	25/14	9.73 ± 1.02	
Sun et al. (40)	EEN+Gln	39	51.71 ± 7.09	25/14	9.73 ± 1.02	G, H
	EEN	39	51.68 ± 7.15	24/15	9.64 ± 1.07	
Chu et al. (27)	EEN+Gln	42	50.62 ± 5.74	27/15	9.76 ± 1.12	G, H, L
	EEN	42	49.48 ± 6.06	26/16	9.63 ± 1.44	
Guan et al. (33)	EEN+Gln	40	58.15 ± 3.13	25/15	Unstates	F, G, I
	EEN	40	58.33 ± 3.2	23/17	Unstates	
Fan et al. (30)	EEN+Gln	46	51.6 ± 3.3	28/18	Unstates	A, B, D, E, F, G
	EEN	46	52.2 ± 2.9	26/20	Unstates	
Yang et al. (48)	EEN+Gln	33	45.51 ± 22.46	17/16	14.03 ± 1.97	B, G, L
	EEN	32	45.52 ± 22.42	16/16	14.17 ± 2.03	

*M/F, Male/Female; TPN, Total Parenteral Nutrition; EEN, Enter Enteral Nutrition; Gln, Glutamine; A, Mortality; B, APACHE II score; C, ICU hospital stay; D, Total length of hospital stay; E, Bloating recovery time; F, Complications; G, Liver function index; H, Kidney function index; I, Inflammatory index; J, Immune index; K, Serum amylase recovery time; L, Response rate*.

*“Total length of hospital stay” refers to the total time spent in hospital for SAP patients, including ICU stay time. “Complications” refers to the number of people with complications from SAP. Regardless of Gln treatment, SAP patients have other diseases caused by disease progression, such as pancreatic pseudocyst, peripancreatic infection, abdominal infection, multiple organ failure, sepsis, and gastrointestinal bleeding. “Response rate” refers to whether it improves the overall treatment effect of severe acute pancreatitis and improves the severity of SAP patients (For example, after 2 weeks of treatment, the patient's symptoms such as abdominal pain and bloating were significantly relieved, and laboratory indicators such as blood/urine amylase, blood routine, liver function, and inflammatory factors were significantly improved)*.

### Risk of Bias Assessment Results

**Figure 2:**
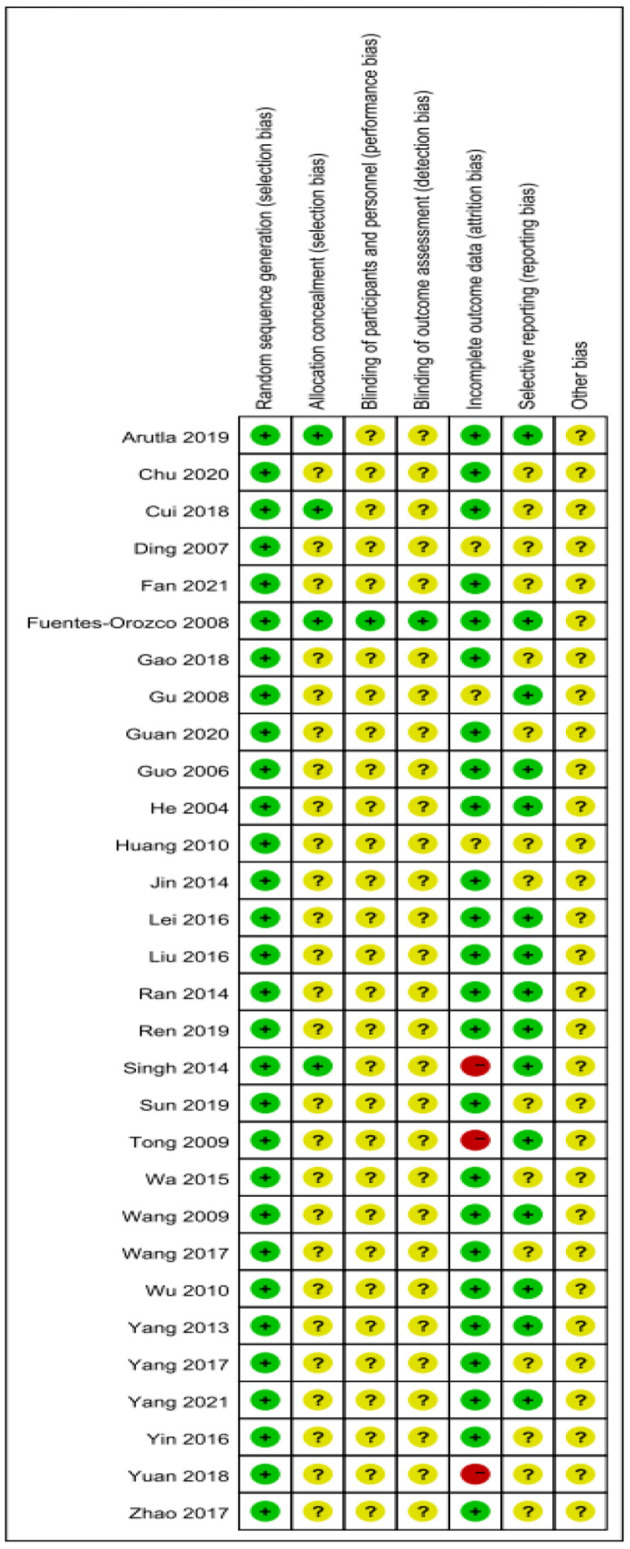
Risk of bias summary.

**Figure 3:**
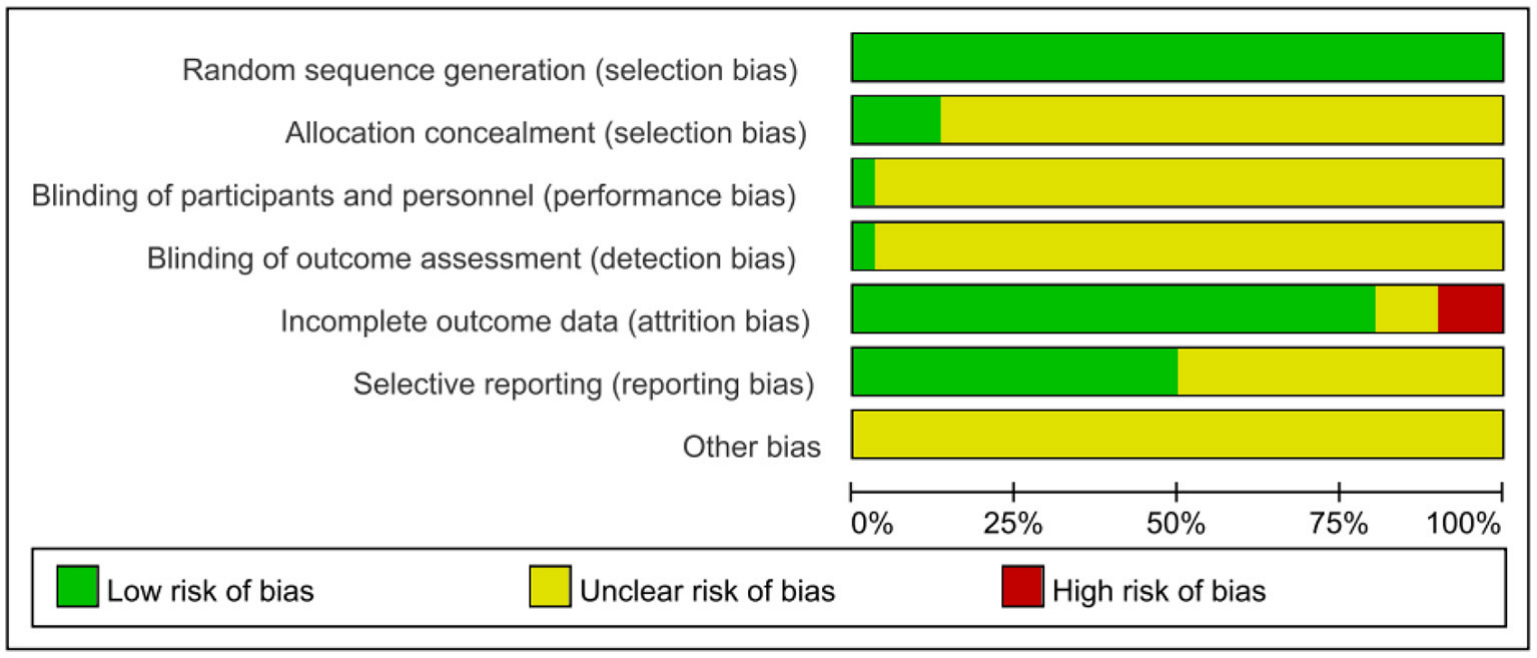
Risk of bias graph.

Given the risk of bias in the published literature, the included studies were analyzed separately for bias to determine the impact on the conclusions. Two authors independently assessed the included RCTs according to the tools of the Cochrane Systematic Review. The quality assessment of the included studies is shown in Figures 2, 3.

### Outcomes of the Intervention

**Figure 4:**
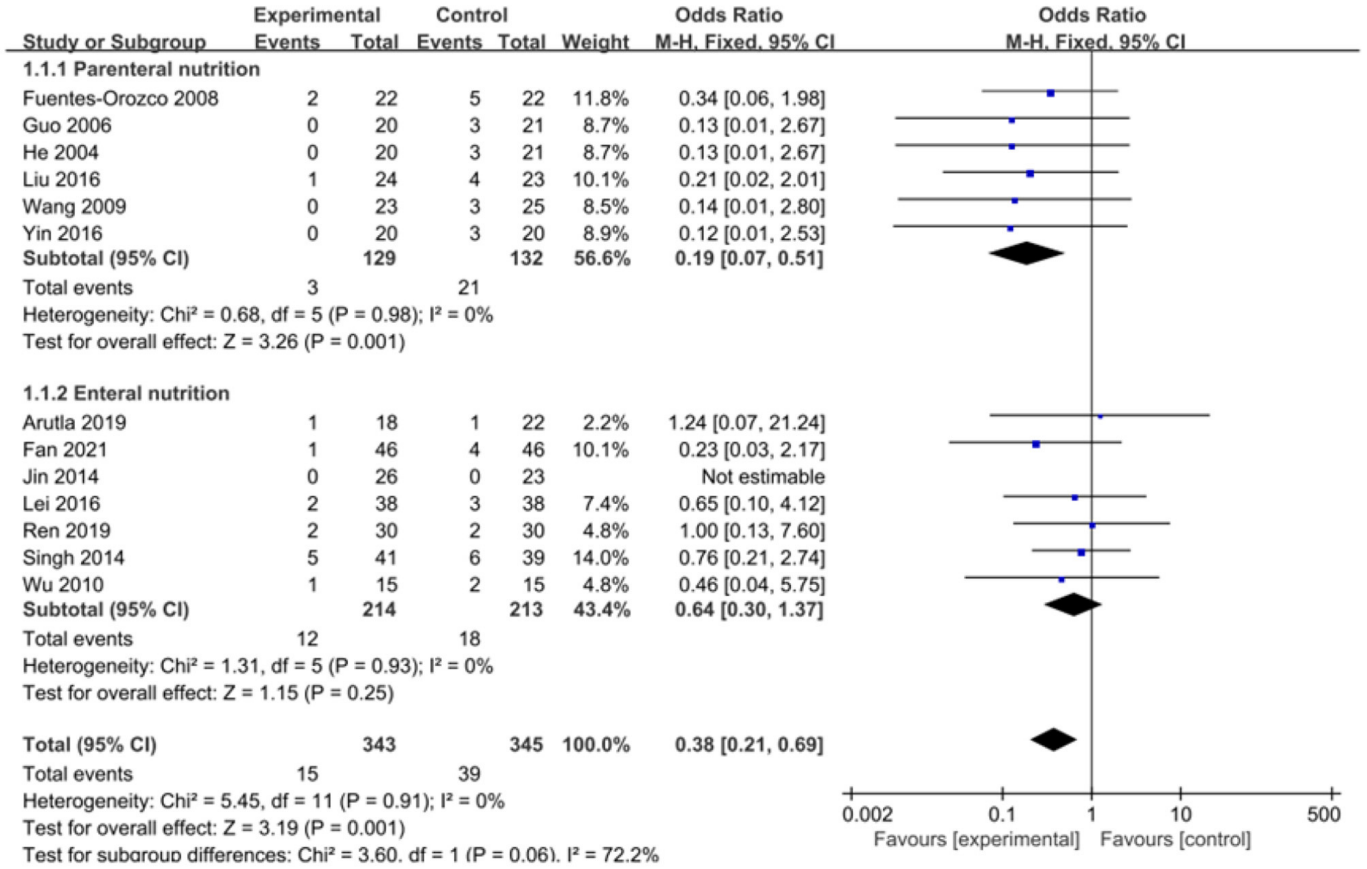
Forest plots of mortality associated with experimental group (parenteral or enteral nutrition group supplemented with Gln) vs. control group (conventional nutrition group). *I*^2^ tests and *P* are the criteria for the heterogeneity test, ♦, pooled odds ratio; —■—, odds ratio, and the edges of ♦, 95% CI.

**Figure 5:**
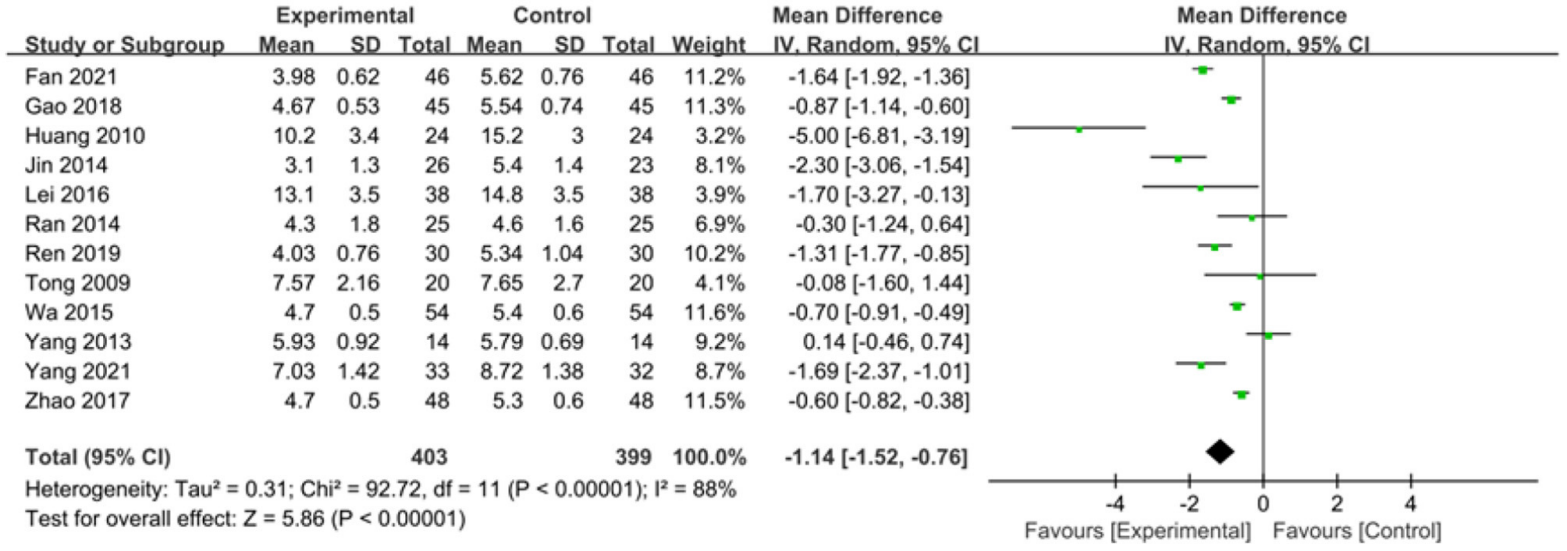
Forest plots of APACHE II score associated with experimental group (parenteral or enteral nutrition group supplemented with Gln) vs. control group (conventional nutrition group). *I*^2^ tests and *P* are the criteria for the heterogeneity test, ♦, pooled mean difference; —■—, mean difference, and the edges of ♦, 95% CI.

**Figure 6:**
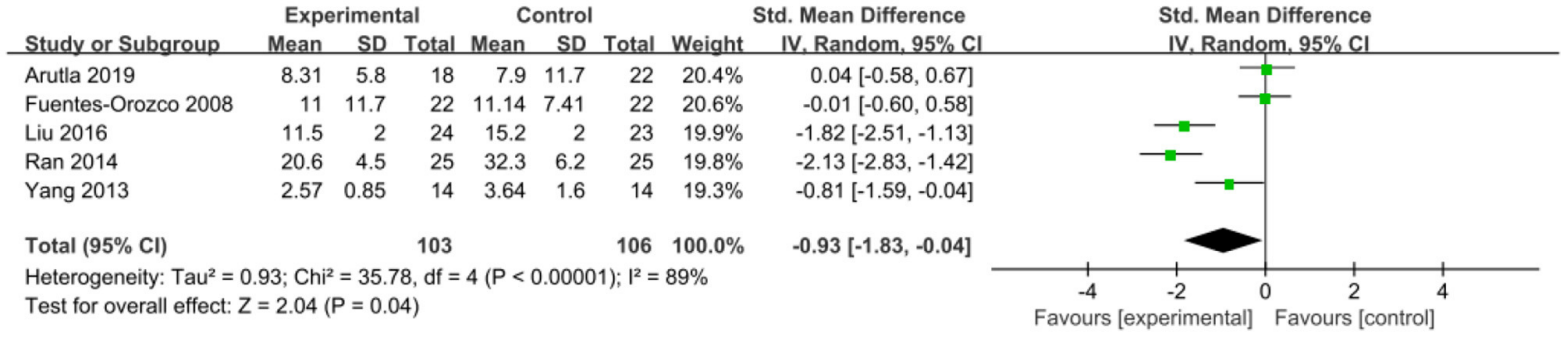
Forest plots of ICU hospital stay associated with experimental group (parenteral or enteral nutrition group supplemented with Gln) vs. control group (conventional nutrition group). *I*^2^ tests and *P* are the criteria for the heterogeneity test, ♦, pooled standard mean difference; —■—, standard mean difference, and the edges of ♦, 95% CI.

**Figure 7:**
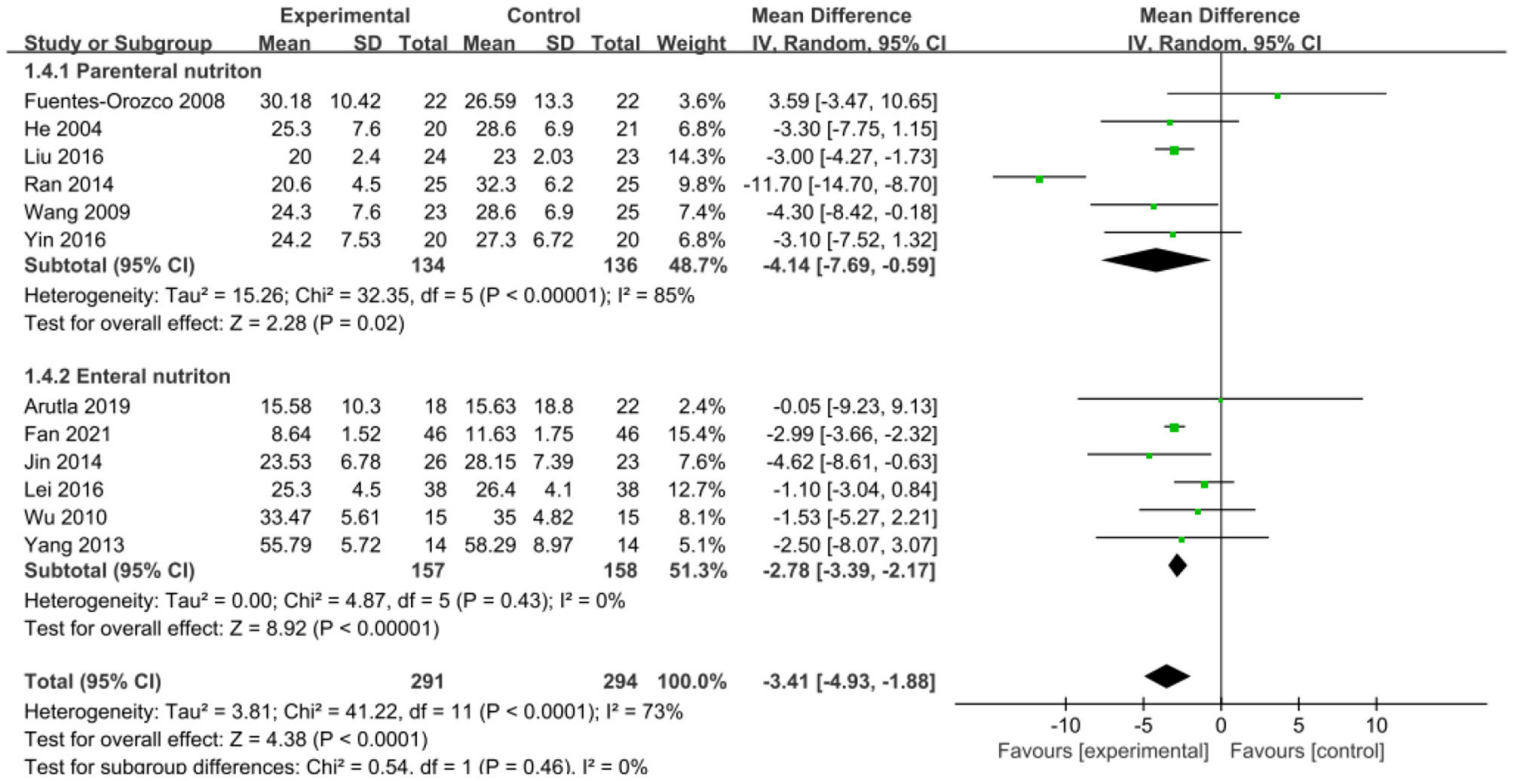
Forest plots of total length of hospital stay associated with experimental group (parenteral or enteral nutrition group supplemented with Gln) vs. control group (conventional nutrition group). *I*^2^ tests and *P* are the criteria for the heterogeneity test, ♦, pooled mean difference; —■—, mean difference, and the edges of ♦, 95% CI.

**Figure 8:**
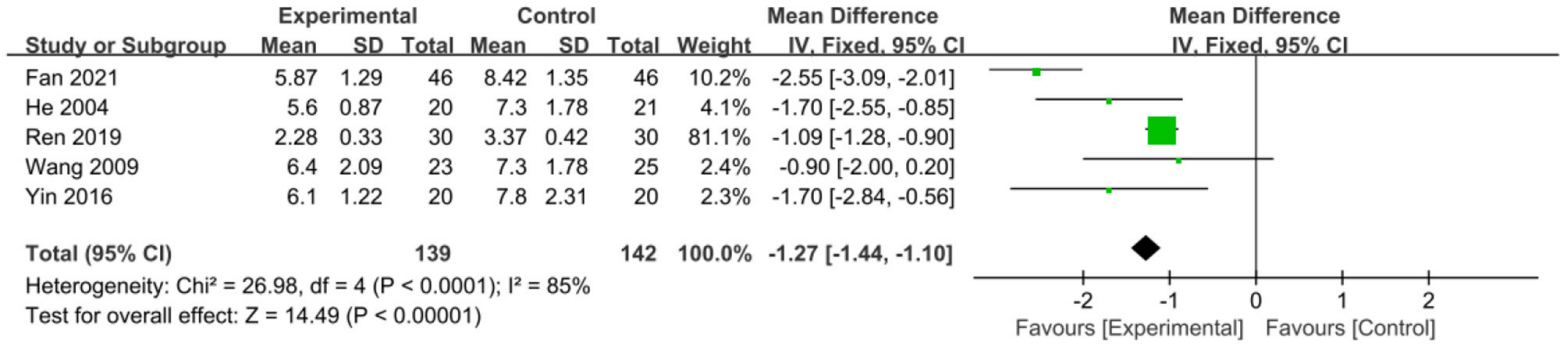
Forest plots of bloating recovery time associated with experimental group (parenteral or enteral nutrition group supplemented with Gln) vs. control group (conventional nutrition group). *I*^2^ tests and *P* are the criteria for the heterogeneity test, ♦, pooled mean difference; —■—, mean difference, and the edges of ♦, 95% CI.

**Figure 9:**
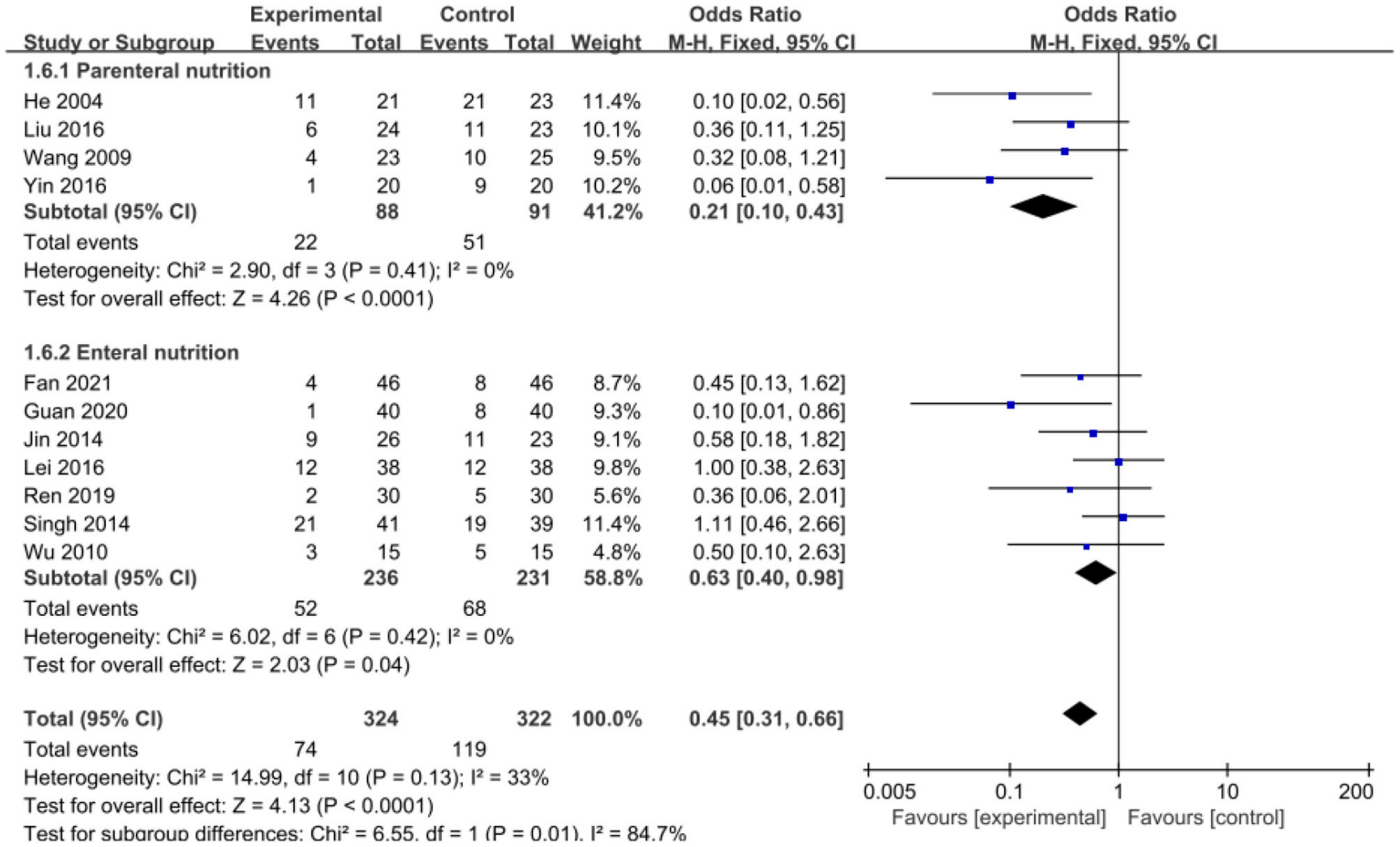
Forest plots of complications associated with experimental group (parenteral or enteral nutrition group supplemented with Gln) vs. control group (conventional nutrition group). *I*^2^ tests and *P* are the criteria for the heterogeneity test, ♦, pooled odds ratio; —■—, odds ratio, and the edges of ♦, 95% CI.

**Figure 10:**
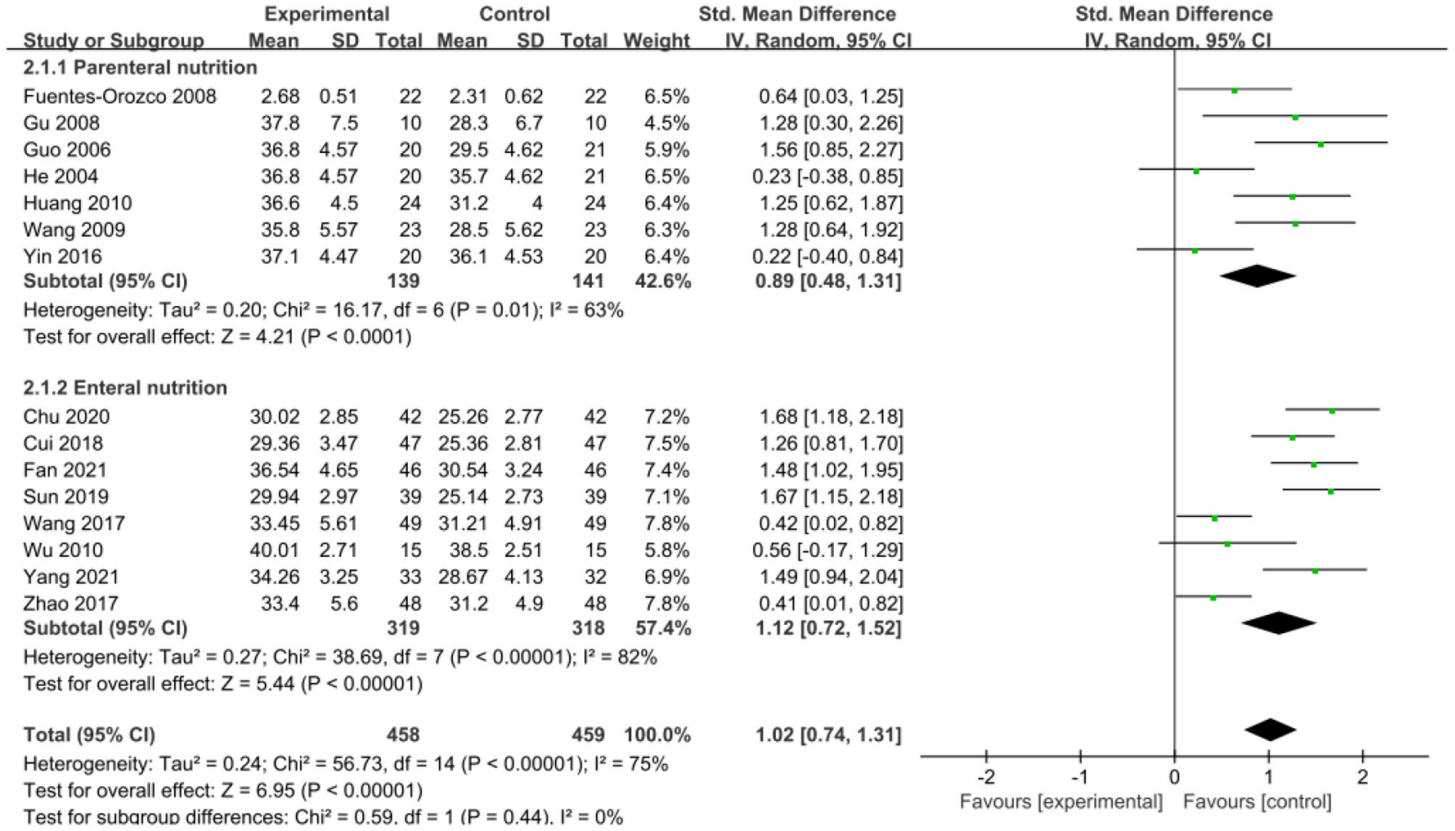
Forest plots of serum albumin associated with experimental group (parenteral or enteral nutrition group supplemented with Gln) vs. control group (conventional nutrition group). *I*^2^ tests and *P* are the criteria for the heterogeneity test, ♦, pooled standard mean difference; —■—, standard mean difference, and the edges of ♦, 95% CI.

**Figure 11:**
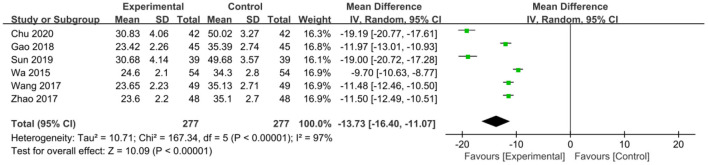
Forest plots of ALT associated with experimental group (parenteral or enteral nutrition group supplemented with Gln) vs. control group (conventional nutrition group). *I*^2^ tests and *P* are the criteria for the heterogeneity test, ♦, pooled mean difference; —■—, mean difference, and the edges of ♦, 95% CI.

**Figure 12:**
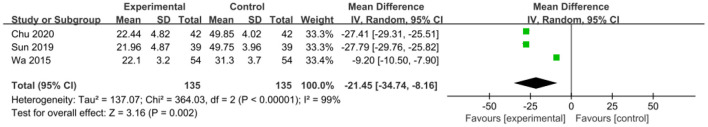
Forest plots of AST associated with experimental group (parenteral or enteral nutrition group supplemented with Gln) vs. control group (conventional nutrition group). *I*^2^ tests and *P* are the criteria for the heterogeneity test, ♦, pooled mean difference; —■—, mean difference, and the edges of ♦, 95% CI.

**Figure 13:**
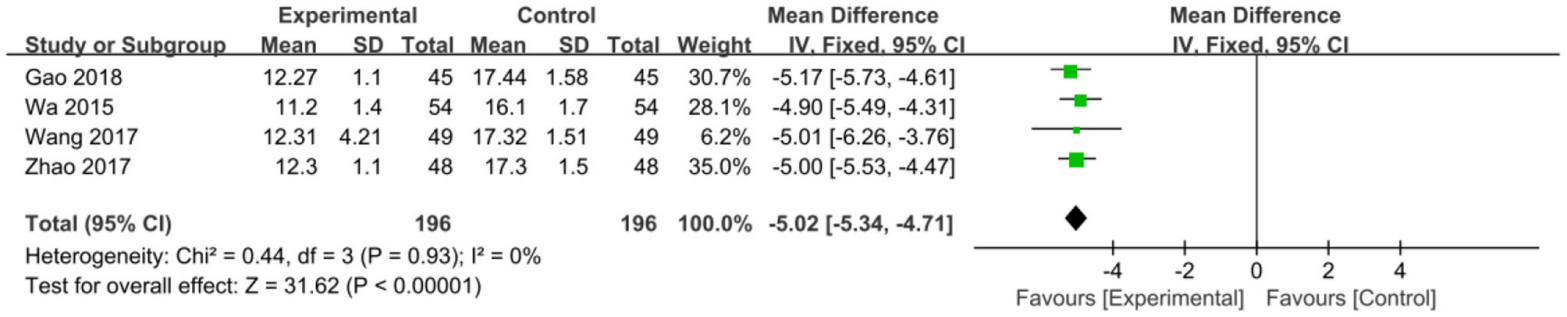
Forest plots of TBIL associated with experimental group (parenteral or enteral nutrition group supplemented with Gln) vs. control group (conventional nutrition group). *I*^2^ tests and *P* are the criteria for the heterogeneity test, ♦, pooled mean difference; —■—, mean difference, and the edges of ♦, 95% CI.

**Figure 14:**
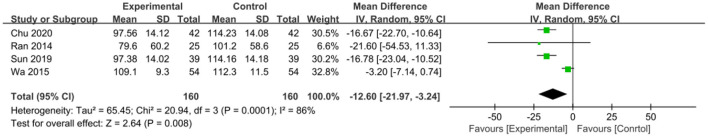
Forest plots of Scr associated with experimental group (parenteral or enteral nutrition group supplemented with Gln) vs. control group (conventional nutrition group). *I*^2^ tests and *P* are the criteria for the heterogeneity test, ♦, pooled mean difference; —■—, mean difference, and the edges of ♦, 95% CI.

**Figure 15:**
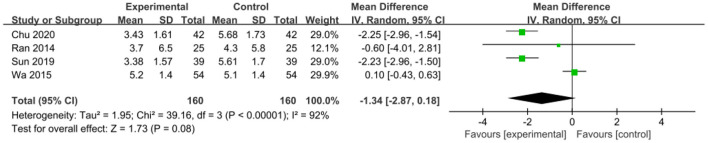
Forest plots of BUN associated with experimental group (parenteral or enteral nutrition group supplemented with Gln) vs. control group (conventional nutrition group). *I*^2^ tests and *P* are the criteria for the heterogeneity test, ♦, pooled mean difference; —■—, mean difference, and the edges of ♦, 95% CI.

**Figure 16:**
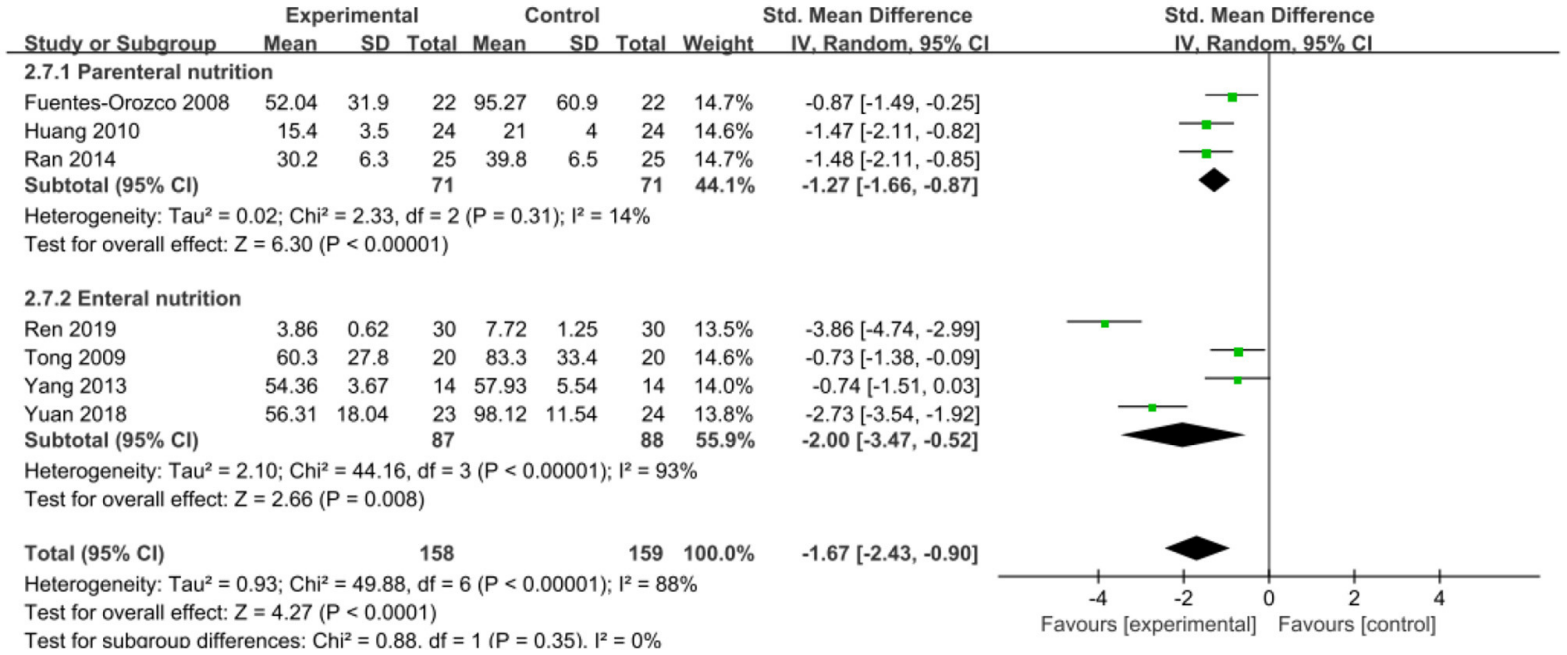
Forest plots of CRP associated with experimental group (parenteral or enteral nutrition group supplemented with Gln) vs. control group (conventional nutrition group). *I*^2^ tests and *P* are the criteria for the heterogeneity test, ♦, pooled standard mean difference; —■—, standard mean difference, and the edges of ♦, 95% CI.

**Figure 17:**
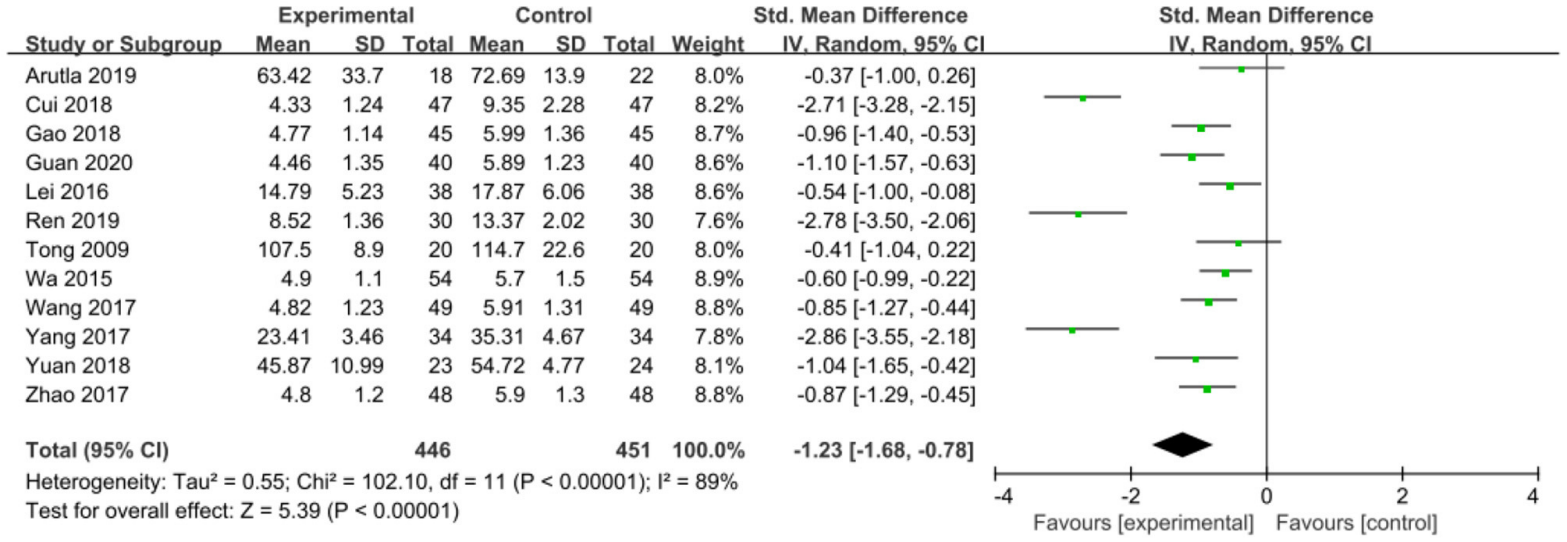
Forest plots of IL-6 associated with experimental group (parenteral or enteral nutrition group supplemented with Gln) vs. control group (conventional nutrition group). *I*^2^ tests and *P* are the criteria for the heterogeneity test, ♦, pooled standard mean difference; —■—, standard mean difference, and the edges of ♦, 95% CI.

**Figure 18:**
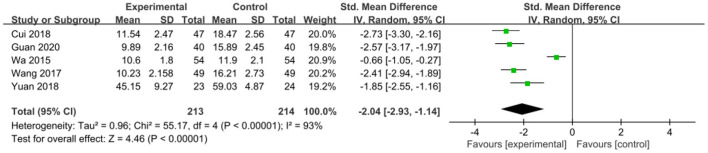
Forest plots of IL-8 associated with experimental group (parenteral or enteral nutrition group supplemented with Gln) vs. control group (conventional nutrition group). *I*^2^ tests and *P* are the criteria for the heterogeneity test, ♦, pooled standard mean difference; —■—, standard mean difference, and the edges of ♦, 95% CI.

**Figure 19:**
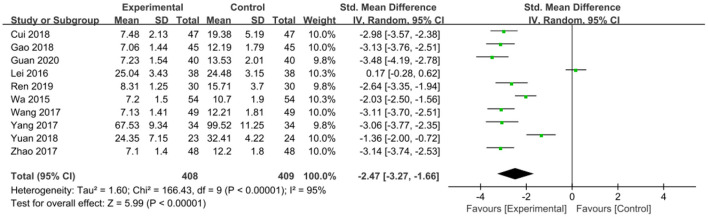
Forest plots of TNF-α associated with experimental group (parenteral or enteral nutrition group supplemented with Gln) vs. control group (conventional nutrition group). *I*^2^ tests and *P* are the criteria for the heterogeneity test, ♦, pooled standard mean difference; —■—, standard mean difference, and the edges of ♦, 95% CI.

**Figure 20:**
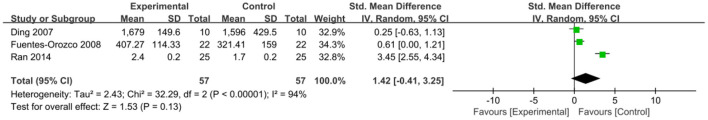
Forest plots of IgA associated with experimental group (parenteral or enteral nutrition group supplemented with Gln) vs. control group (conventional nutrition group). *I*^2^ tests and *P* are the criteria for the heterogeneity test, ♦, pooled standard mean difference; —■—, standard mean difference, and the edges of ♦, 95% CI.

**Figure 21:**
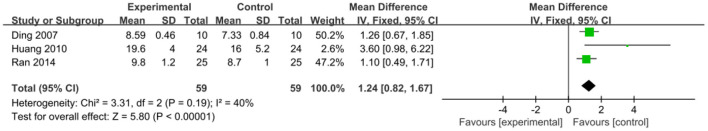
Forest plots of IgG associated with experimental group (parenteral or enteral nutrition group supplemented with Gln) vs. control group (conventional nutrition group). *I*^2^ tests and *P* are the criteria for the heterogeneity test, ♦, pooled mean difference; —■—, mean difference, and the edges of ♦, 95% CI.

**Figure 22:**
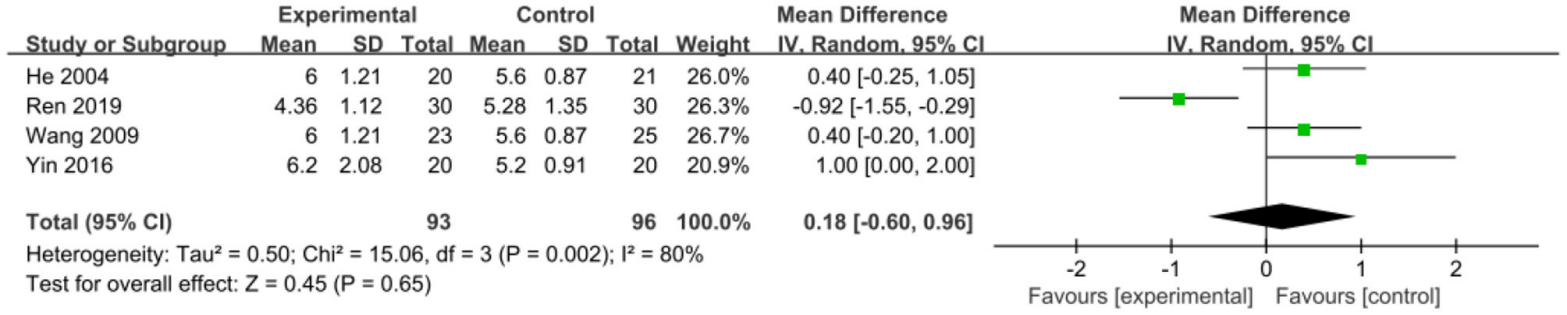
Forest plots of serum amylase recovery time associated with experimental group (parenteral or enteral nutrition group supplemented with Gln) vs. control group (conventional nutrition group). *I*^2^ tests and *P* are the criteria for the heterogeneity test, ♦, pooled mean difference; —■—, mean difference, and the edges of ♦, 95% CI.

**Figure 23:**
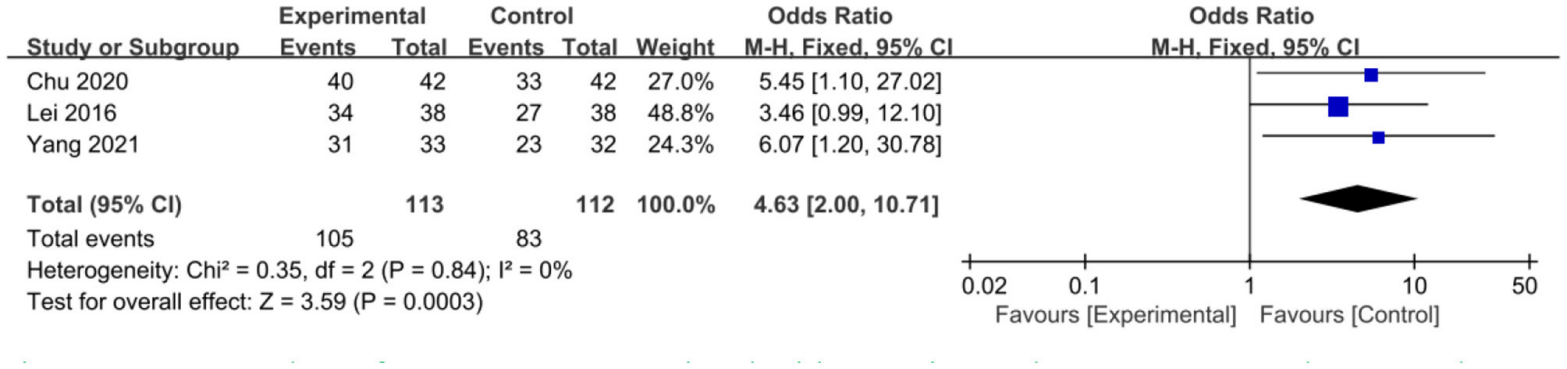
Forest plots of response rate associated with experimental group (parenteral or enteral nutrition group supplemented with Gln) vs. control group (conventional nutrition group). *I*^2^ tests and *P* are the criteria for the heterogeneity test, ♦, pooled odds ratio; —■—, odds ratio, and the edges of ♦, 95% CI.

The intervention outcomes were divided into primary and secondary outcomes; the primary outcomes included mortality (Figure 4), APACHE II score (Figure 5), ICU hospital stay (Figure 6), total length of hospital stay (Figure 7), bloating recovery time (Figure 8) and complications (Figure 9), and secondary outcomes included liver function indicators (Figures 10–13), kidney function indicators (Figures 14, 15), inflammatory indicators (Figures 16–19), immune indicators (Figures 20, 21), serum amylase recovery time (Figure 22), and response rate (Figure 23). In Figures 4–23, “experimental” represents the parenteral or enteral nutrition group supplemented with Gln, and “control” means the conventional nutrition group. Rhombuses in the forest plot represent the results of the meta-analysis; the center of the rhombuses represents the point estimates of the effect size of the summary results, such as the OR value, MD value and SMD value, and the width of the rhombuses represents the 95% CIs of the effect size of the pooled results.

#### Primary Outcomes

##### Mortality

The effect of adding Gln on the mortality of SAP patients was repored in 13 included studies (*n* = 688 patients). A fixed-effects model was selected since no significant heterogeneity was present among the studies (*P* = 0.91, *I*^2^ = 0%). The pooled results showed that Gln supplementation significantly reduced patient' mortality (OR = 0.38, 95% CI: 0.21–0.69, *P* = 0.001; Figure 4). We performed an additional subgroup analysis by stratifying parenteral nutrition vs. enteral nutrition. Compared with the conventional nutrition group, enteral nutrition with Gln failed to effectively reduce the mortality of SAP patients (OR = 0.64, 95% CI: 0.30–1.37, *P* = 0.25), while parenteral Gln supplementation could significantly reduce patient mortality (OR = 0.19, 95% CI: 0.07–0.51, *P* = 0.001).

##### APACHE II Score

Twelve studies involving 802 patients reported the APACHE II score outcome. Given that significant heterogeneity was present among these studies (*P* < 0.00001, *I*^2^ = 88%), a random-effects model was used. The pooled results indicated that Gln supplementation effectively reduced the APACHE II score compared to the conventional nutrition group (SMD = −1.14, 95% CI: −1.52 to −0.76, *P* < 0.00001; Figure 5).

##### ICU Hospital Stay

ICU hospital stay was reported in five included studies involving 209 patients. There was significant heterogeneity among these studies (*P* < 0.00001, *I*^2^ = 89%), which was not reduced after removing one study at a time for sensitivity analysis; accordingly, a random-effects model was selected. The pooled results indicated that Gln supplementation was associated with a significantly shorter ICU hospital stay (SMD = −0.93, 95% CI: −1.83 to −0.04, *P* = 0.04; Figure 6).

##### Total Length of Hospital Stay

An association between the total length of hospital stay and Gln supplementation was reported in twelve studies involving 585 patients. There was significant heterogeneity between these studies (*P* < 0.0001, *I*^2^ = 73%), and, a random-effects model was applied. The pooled results showed that Gln supplementation significantly reduced the total length of hospital stay (MD = −3.41, 95% CI: −4.93 to −1.88, *P* < 0.0001; Figure 7). After subgroup analysis by stratifying enteral vs. parenteral supplementation, the modalities of Gln supplementation and the inclusion criteria of the Ran et al. study (32) were found to be the main source of heterogeneity. Furthermore, subgroup analysis showed both parenteral (MD = −4.14, 95% CI: −7.69 to −0.59, *P* = 0.02) and enteral supplementation (MD = −2.78, 95% CI: −3.39 to −2.17, *P* < 0.00001) can effectively reduce the total hospitalization time of patients compared with the conventionally nutrition group.

##### Bloating Recovery Time

To compare the effect of Gln on bloating recovery time in patients, five studies involving a total of 281 patients were included. There was significant heterogeneity among these studies (*P* < 0.0001, *I*^2^ = 85%), and a random-effects model was used. The pooled results showed that Gln supplementation significantly shortened the bloating recovery time in patients (MD = −1.27, 95% CI: −1.44 to −1.10, *P* < 0.00001; Figure 8).

##### Complications

Eleven included studies involving 646 patients reported complications as an outcome, involving 646 patients. No significant heterogeneity was found among these studies (*P* = 0.13, *I*^2^ = 33%). The pooled rates showed that Gln supplementation significantly reduced the incidence of complications in patients (OR = 0.45, 95% CI: 0.31–0.66, *P* < 0.0001; Figure 9). A subgroup analysis showed that compared with the conventional nutrition group, Gln parenteral supplementation (OR = 0.21, 95% CI: 0.10–0.43, *P* < 0.0001), and enteral nutrition could significantly reduce complications in SAP patients (OR = 0.63, 95% CI: 0.40–0.98, *P* = 0.04), and no significant heterogeneity was found among the included studies (*I*^2^ = 0).

#### Secondary Outcomes

##### Liver Function Indicators

*Serum albumin*. An association between serum albumin and Gln supplementation was reported in 15 included studies (*n* = 917). The pooled estimates showed that Gln supplementation significantly increased serum albumin level (SMD = 1.02, 95% CI: 0.74–1.31, *P* < 0.00001; Figure 10). However, there was significant heterogeneity among these studies (*P* < 0.00001, *I*^2^ = 75%). Substratification into parenteral and enteral subgroups failed to reduce the heterogeneity, suggesting that Gln supplementation was not a source of heterogeneity. Subgroup analysis showed that parenteral (SMD = 0.89, 95% CI: 0.48–1.31, *P* < 0.0001) and enteral Gln supplementation significantly increased serum albumin level (SMD = 1.12, 95% CI: 0.72–1.52, *P* < 0.00001) compared with the conventional nutrition group.

*ALT*. Six studies reported ALT for the two groups, involving 554 patients with severe pancreatitis, and there was significant heterogeneity among these studies (*P* < 0.00001, *I*^2^ = 97%). After conducting a sensitivity analysis, the heterogeneity was not reduced. Accordingly, the heterogeneity was attributed to differences in detection instruments (Supplementary Material 1). We selected a random-effects model, and the pooled estimates suggested that Gln supplementation significantly reduced ALT level (MD = −13.73, 95% CI: −16.40 to −11.07, *P* < 0.00001; Figure 11).

*AST*. AST was reported in 3 included studies involving a total of 270 patients. A random-effects model was used since significant heterogeneity was observed among these studies (*P* < 0.00001, *I*^2^ = 99%). The meta-analysis results showed that Gln supplementation was more effective in reducing AST level (MD = −21.45, 95% CI: −34.74 to −8.16, *P* = 0.002; Figure 7).

*TBIL*. Four studies with a total of 392 patients reported TBIL. There was little heterogeneity among these studies (*P* = 0.93, *I*^2^ = 0%), and a fixed-effects model was used. The pooled results suggested that Gln supplementation significantly reduced TBIL level (MD = −5.02, 95% CI: −5.34 to −4.71, *P* < 0.00001; Figure 13).

##### Kidney Function Indicators

*Scr*. Four studies involving 320 patients were included to assess the effect of Gln supplementation on Scr levels. Significant heterogeneity was observed among these studies (*P* = 0.0001, *I*^2^ = 86%) and a random-effects model was selected. The pooled results suggested that Gln addition was effective in reducing Scr levels (MD = −12.60, 95% CI: −21.97 to −3.24, *P* = 0.008; Figure 14).

*BUN*. Four studies, including 320 patients, reported BUN results. There was significant heterogeneity among these studies (*P* < 0.00001, *I*^2^ = 92%) and a random-effects model was used. The combined results indicated that the addition of Gln was not significantly correlated with BUN levels (MD = −1.34, 95% CI: −2.87 to 0.18, *P* = 0.08; Figure 15).

##### Inflammatory Indicators

*CRP*. Seven studies involving a total of 317 patients reported this outcome. There was large heterogeneity among these studies (*P* < 0.00001, *I*^2^ = 88%), and the pooled estimates suggested that Gln supplementation effectively reduced CRP levels in SAP patients (SMD = −1.67, 95% CI: −2.43 to −0.90, *P* < 0.0001; Figure 16). After substratification into parenteral and enteral group, we found that the feeding route was a source of heterogeneity. Our subgroup analysis showed that compared with the conventional nutrition group, both parenteral (SMD = −1.27, 95% CI: −1.66 to −0.87, *P* < 0.00001) and enteral Gln significantly reduced CRP levels (SMD = −2.00, 95% CI: −3.47 to −0.52, *P* = 0.008).

*IL-6*. IL-6 was reported in 12 studies involving 897 patients. There was significant heterogeneity among these studies (*P* < 0.00001, *I*^2^ = 89%) and a random-effects model was used. The results indicated that Gln supplementation effectively reduced IL-6 levels (SMD = −1.23, 95% CI: −1.68 to −0.78, *P* < 0.00001; Figure 17).

*IL-8*. To compare the effects on IL-8 levels in patients, five studies involving 427 patients were included. There was significant heterogeneity among these studies (*P* < 0.00001, *I*^2^ = 93%), and a random-effects model was used. The pooled results suggested that Gln supplementation effectively reduced IL-8 level (SMD = −2.04, 95% CI: −2.93 to −1.14, *P* < 0.00001; Figure 18).

*TNF-α*. To compare the effect of Gln supplementation on TNF-α level in SAP patients, 10 studies involving 817 patients were included. Given that significant heterogeneity was observed among these studies (*P* < 0.00001, *I*^2^ = 95%), we applied a random-effects model. The combined results showed that nutritional supplementation with Gln was effective in reducing TNF-α levels (SMD = −2.47, 95% CI: −3.27 to −1.66, *P* < 0.00001; Figure 19).

##### Immune Indicators

*IgA*. IgA was reported in three studies involving 114 patients. Given that there was significant heterogeneity among these studies (*P* < 0.00001, *I*^2^ = 94%), a random-effects model was selected. The results indicated no statistically significant difference in IgA levels between the Gln supplementation group and the conventional nutrition group (SMD = 1.42, 95% CI: −0.41 to 3.25, *P* = 0.13; Figure 20).

*IgG*. This outcome was reported in 3 included studies involving 118 patients. A fixed-effects model was used since no significant heterogeneity was observed among these studies (*P* = 0.19, *I*^2^ = 40%). The pooled estimates suggested that Gln supplementation can significantly increase IgG levels (MD = 1.24, 95% CI: 0.82–1.67, *P* < 0.00001; Figure 21).

##### Serum Amylase Recovery Time

Serum amylase recovery time was included in four studies, involving 118 patients. There was significant heterogeneity among these studies (*P* = 0.002, *I*^2^ = 80%) and a random-effects model was used. The pooled results indicated no significant difference in serum amylase recovery time between treatment with or without Gln supplementation (MD = 0.18, 95% CI: −0.60 to 0.96, *P* = 0.65; Figure 22).

##### Response Rate

Three included studies involving 225 patients reported the response rate. Little heterogeneity was observed among these studies (*P* = 0.84, *I*^2^ = 0%), and, a fixed-effects model was used. The pooled results showed that the response rate between Gln-supplementation and the control group was statistically significant (OR = 4.63, 95% CI: 2.00–10.71, *P* = 0.0003; Figure 23).

### Sensitivity Analysis

Sensitivity analyses were performed by omitting one study at a time. During analysis of kidney function indicators, when the study of Wa et al. (36) was excluded, the pooled estimates showed that Gln supplementation was more effective in reducing BUN levels than the control group (MD: −2.20, 95%CI, −2.71 to −1.70, *P* < 0.00001), which was inconsistent with the preliminary results and significantly reduced heterogeneity (*I*^2^ = 0%). In terms of serum amylase recovery time, when we excluded the study by Ren et al. (33), the pooled results showed that Gln supplementation failed to effectively reduce the serum amylase recovery time (MD: 0.50; 95% CI, 0.10–0.90; *P* < 0.00001), but the heterogeneity (*I*^2^ = 0%) was significantly reduced.

### Publication Bias

**Figure 24:**
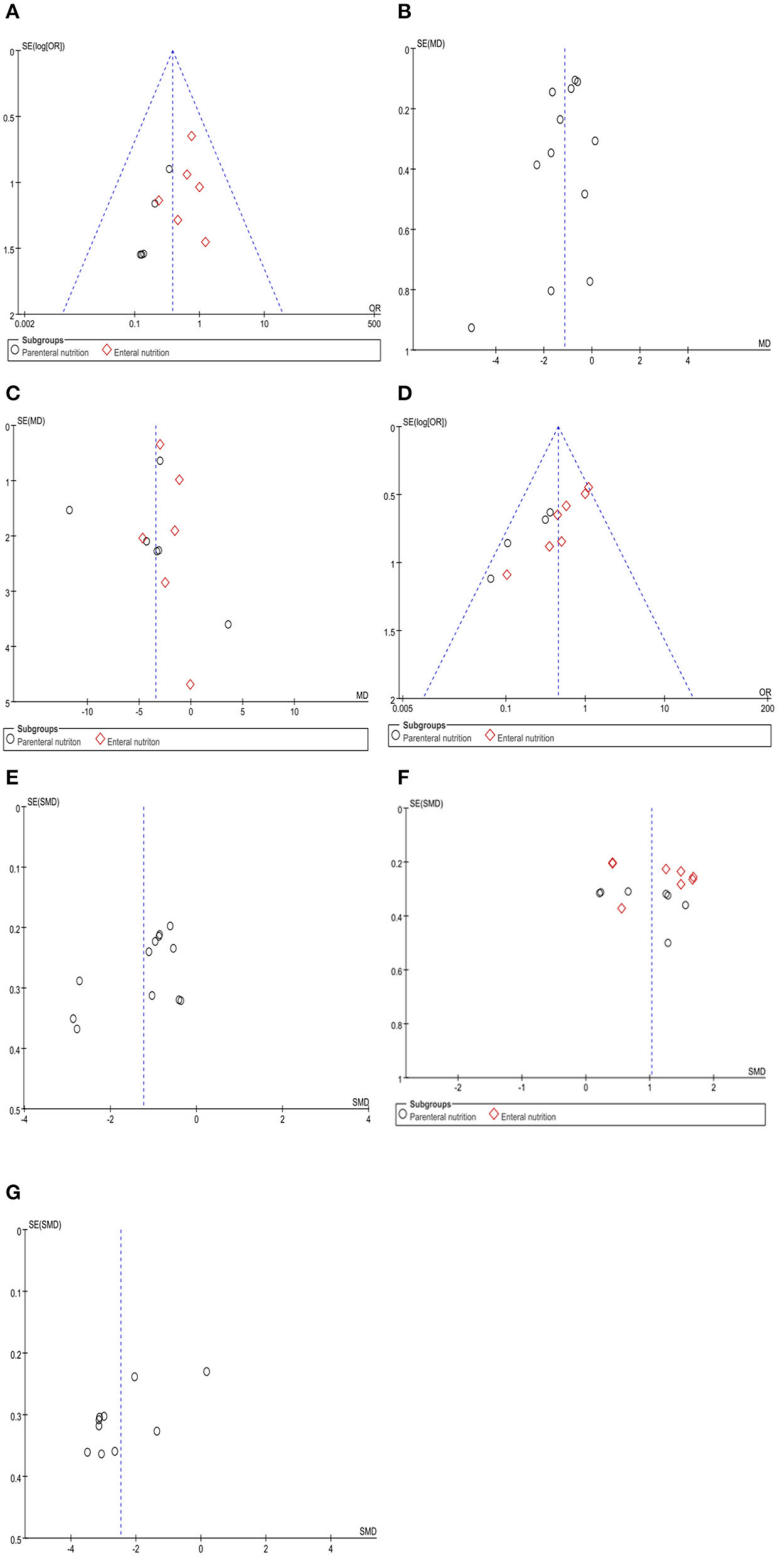
Funnel plots of the included studies for mortality **(A)**, APACHE II score **(B)**, total length of hospital stay **(C)**, complications **(D)**, serum albumin **(F)**, IL-6 **(E)**, and TNF-α **(G)**.

Funnel plot and Egger's test were used to evaluate publication bias of mortality, APACHE II score, complications, total length of hospital stay, serum albumin, IL-6, and TNF-α. The results showed significant publication bias for mortality (Egger's test, *P* = 0.025), complications (Egger's test, *P* = 0.000), and TNF-α (Egger's test, *P* = 0.020). However, no significant publication bias was observed for in the APACHE II score (Egger's test, *P* = 0.262), total length of hospital stay (Egger's test, *P* = 0.892), serum albumin (Egger's test, *P* = 0.545), and IL-6 (Egger's test, *P* = 0.059) (Figure 24).

## Discussion

The meta-analysis aimed to evaluate the efficacy of Gln parenteral or enteral nutrition vs. conventional nutrition in patients with SAP. Outcomes, such as mortality, complications, the hospital stay of SAP patients, and other test results, were evaluated.

Consistent with the literature, we found that nutritional support with Gln may be an effective therapeutic approach for SAP. Importantly, unlike enteral Gln supplementation (OR = 0.64, 95% CI: 0.30–1.37, *P* = 0.25), parenteral nutrition with Gln effectively reduced mortality (OR = 0.19, 95% CI: 0.07–0.51, *P* = 0.001), which was inconsistent with the study by Jiang et al. (14) who reported that enteral Gln supplementation could significantly reduce the mortality risk (risk ratio= 0.38; 95% CI, 0.16–0.90; *p* = 0.03). Accordingly, future clinical studies are warranted to validate this finding. Regarding the total length of hospital stay and complications, both parenteral and enteral Gln supplementation were more effective than conventional nutrition (*P* < 0.05). Interestingly, we found that when a Gln concentration of 0.4 g/kg used in 17 included studies (6, 12, 27, 28, 30, 31, 33–35, 37, 40, 42–45, 48, 49, 51) for parenteral supplementation did not reduce the total hospital stay (*P* > 0.05), compared to enteral supplementation (*P* < 0.05; Supplementary Material 3). However, parenteral Gln supplementation was more effective than enteral supplementation in reducing the incidence of complications (Supplementary Material 3). In terms of APACHE II score, ICU hospital stay and bloating recovery time, Gln supplementation was more effective than conventional nutrition (*P* < 0.05). Nonetheless, given the limited number of included studies, subgroup analysis could not be performed. 0.4 g/kg Gln yield results, emphasizing the need for future research with large sample data. As for secondary outcomes, in terms of liver function indicators, nutritional support therapy with Gln effectively improved liver function (*P* < 0.05). Moreover, we found that Gln supplementation (enteral or parenteral nutrition) increased serum albumin levels. We further analyzed the pros and cons of different methods of 0.4 g/kg Gln supplementation and found that parenteral supplementation was inferior to as enteral supplementation (Supplementary Material 3). Interestingly, compared with the conventional nutrition group, Gln supplementation could effectively reduce Scr levels (MD = −12.60, 95% CI: −21.97 to −3.24, *P* = 0.008). However, BUN results were not consistent with the above findings (MD = −1.34, 95% CI: −2.87 to 0.18, *P* = 0.08), and this discrepancy may be attributed to the quality of the included literature. Gln supplementation was more effective than conventional nutrition in reducing the inflammatory indicators, including CRP, IL-6, IL-8, and TNF-α, and could effectively alleviate the inflammatory state in SAP. However, in terms of immune indicators, Gln therapy significantly improved IgG levels (MD = 1.24, 95% CI: 0.82–1.67, *P* < 0.00001), but no statistically significant differences in IgA were observed (SMD = 1.42, 95% CI: −0.41 to 3.25, *P* = 0.13). In addition, no significant difference in serum amylase recovery time was observed between the two groups (*P* > 0.05).

In this meta-analysis, Gln supplementation was effective for all parameters except BUN, IgA and serum amylase recovery time. During the subgroup analysis, except for mortality, the nutrition route did not affect the outcome of corresponding indicators. At the similar Gln concentrations (0.4 g/kg), the two supplementation methods did not change mortality, suggesting that parenteral supplementation was more effective for SAP patients. Indeed, notwithstanding that parenteral Gln supplementation was inferior to enteral supplementation in terms of total hospital stay and serum albumin levels, parenteral supplementation was associated with a lower incidence of complications in SAP patients. Although some biochemical parameters, such as CRP, serum albumin and Scr, improved with Gln use, there were no significant changes in primary outcomes, such as mortality (with enteral supplementation). It has been established that the systemic inflammatory response in AP causes an increase in calorie and protein metabolism, which leads to systemic organ damage and substantial nutrient loss, and long-term fasting aggravates the negative nitrogen balance (54). Inflammatory storms and inadequate nutrient intake can exacerbate organ damage and intestinal dysfunction. Moreover, peripancreatic infection and necrosis cause severe sepsis, further disrupting the intestinal barrier and leading to high patient mortality. Therefore, protecting the integrity of the intestinal barrier is critical for reducing patient mortality and improving primary clinical outcomes. In the present study, we found that enteral Gln supplementation did not improve the mortality of SAP patients, which may be attributed to the non-recovery of the intestinal function, and intestinal absorption of Gln was significantly lower than that of intravenous infusion. For patients with very severe symptoms, Gln yielded no significant effects in the short term. In addition, although the effect of enteral supplementation of Gln can be effective, it is still inferior to parenteral nutrition, which is mainly related to whether Gln is effectively utilized during early stages. The current study found that parenteral supplementation was more effective than enteral supplementation on primary and secondary outcomes. However, a limited number of studies were included, and the severity of SAP patients and the timing, dose, and duration of Gln use remain primarily understudied, warranting further studies.

Our study findings are consistent with previous meta-analyses (14, 22). Seven RCTs were included in the meta-analysis by Jiang et al. (14), with a relatively smaller number of patients and outcomes involved. A meta-analysis by Li et al. (22), which included 10 RCTs showed that compared with enteral nutrition (SMD = 0.36, 95% CI: −0.08 to 0.80, *P* > 0.05), intravenous infusion Gln was more effective in reducing plasma albumin levels (SMD = 1.19, 95% CI: 0.62–1.77, *P* < 0.05), which was inconsistent with our study, and different results were also observed with CRP In the present meta-analysis, 30 RCTs were included based on strict inclusion and exclusion criteria with increased sample size, primary and secondary outcomes. We did not analyze indicators with <3 included studies to reduce the risk of bias and ensure the robustness and accuracy of our findings. Morever, we searched eight major databases to ensure that relevant articles were not missed. Finally, we performed a literature quality assessment, sensitivity analysis, and publication bias detection on the included literature and presented the results in forest and funnel plots for a comprehensive meta-analysis.

One strength of this study is that it summarizes and analyzes the latest related literature, which makes up for the knowledge gap in this research field. In addition, we included a relatively large number of RCTs and a large sample size, providing robust evidence for our findings. However, some limitations of our study warrant attention. First of all, some indicators such as BUN were reported in few studies which may be a source of publication bias. Moreover, the reliability of our findings was affected due to differences in detection method. Given the presence of high heterogeneity among the included studies, a random-effects model was used to improve the stability of these outcomes. Finally, since most of the included studies originated from China, the present study's findings cannot be generalized to other regions, emphasizing the need for more studies worldwide.

## Conclusion

The findings of this study provided compelling evidence that nutritional support therapy with Gln is an effective therapy for SAP, especially for the recovery of relevant biochemical indicators of patients during hospitalization and the reduction of hospitalization time. Subgroup analysis found that parenteral nutrition supplementation with Gln was more likely to reduce mortality and complications in patients, so parenteral nutrition seemed to be a better choice for patients with severe symptoms. However, there are few studies on the timing and dose of Gln supplementation in SAP patients, and prospective trials are needed to prove it. In addition, the outcome of Gln nutrition therapy for some indicators is largely unclear, such as serum amylase and intestinal function recovery time, whether to transfer to surgery, etc. Safety is also the focus of research, including gastrointestinal reactions, metabolic disorders, but it has not been paid attention to in detail in previous studies. In the implementation of medical decisions, clinicians should weigh the patient's condition based on the above factors, so as to facilitate a good prognosis of patients. Further research with larger sample size is needed to improve the current understanding of these clinical outcomes and accurately evaluate the efficacy of nutritional therapy with Gln for SAP patients.

## Data Availability Statement

The original contributions presented in the study are included in the article/Supplementary Material, further inquiries can be directed to the corresponding author.

## Author Contributions

SD: writing—original draft preparation, supervision, and project administration. ZZ: writing—original draft preparation and visualization. XL, ZC, and WJ: writing—review and editing and supervision. WZ: writing—original draft preparation, supervision, project administration, and funding acquisition. All authors have read and agreed to the published version of the manuscript.

## Funding

This work was supported by the Science and Technology Projects of Chengguan District in Lanzhou [grant number 2020-2-11-4] and Traditional Chinese Medicine Scientific Research Project of Gansu Province, China [grant number GZKP-2020-28].

## Conflict of Interest

The authors declare that the research was conducted in the absence of any commercial or financial relationships that could be construed as a potential conflict of interest.

## Publisher's Note

All claims expressed in this article are solely those of the authors and do not necessarily represent those of their affiliated organizations, or those of the publisher, the editors and the reviewers. Any product that may be evaluated in this article, or claim that may be made by its manufacturer, is not guaranteed or endorsed by the publisher.

## Abbreviations

SAP: severe acute pancreatitis
Gln: glutamine
RCTs: randomized controlled trials
Scr: serum creatinine
CRP: C-reaction protein
AP: acute pancreatitis
MOF: multisystem organ failure
MODS: multiple organ dysfunction syndromes
RR: relative risk
MD: mean difference
SMD: standard mean difference
ALT: alanine aminotransferase
AST: aspartate aminotransferase
TBIL: total bilirubin
BUN: blood urea nitrogen
IL-6: interleukin- 6
TNF-α: tumor necrosis factor α.
