# Supplementary material for: Efficacy of Glutamine in Treating Severe Acute Pancreatitis: A Systematic Review and Meta-Analysis
Source: Front Nutr. 2022 Jun 14;9:865102. doi: 10.3389/fnut.2022.865102 (PMC9237617; doi:10.3389/fnut.2022.865102)
Supplement: Supplementary file 1 [file Data_Sheet_1.PDF]

PRISMA 2009 Checklist

Section/Topic	Item No	Checklist item	Reported on page No
**TITLE**			
Title	1	Identify the report as a systematic review, meta-analysis, or both	1
**ABSTRACT**			
Structured summary	2	Provide a structured summary including, as applicable, background, objectives, data sources, study eligibility criteria, participants, interventions, study appraisal and synthesis methods, results, limitations, conclusions and implications of key findings, systematic review registration number	1
**INTRODUCTION**			
Rationale	3	Describe the rationale for the review in the context of what is already known	2
Objectives	4	Provide an explicit statement of questions being addressed with reference to participants, interventions, comparisons, outcomes, and study design (PICOS)	4
**METHODS**			
Protocol and registration	5	Indicate if a review protocol exists, if and where it can be accessed (such as web address), and, if available, provide registration information including registration number	4
Eligibility criteria	6	Specify study characteristics (such as PICOS, length of follow-up) and report characteristics (such as years considered, language, publication status) used as criteria for eligibility, giving rationale	5
Information sources	7	Describe all information sources (such as databases with dates of coverage, contact with study authors to identify additional studies) in the search and date last searched	5
Search	8	Present full electronic search strategy for at least one database, including any limits used, such that it could be repeated	5
Study selection	9	State the process for selecting studies (that is, screening, eligibility, included in systematic review, and, if applicable, included in the meta-analysis)	5
Data collection process	10	Describe method of data extraction from reports (such as piloted forms, independently, in duplicate) and any processes for obtaining and confirming data from investigators	6
Data items	11	List and define all variables for which data were sought (such as PICOS, funding sources) and any assumptions and simplifications made	6
Risk of bias in individual studies	12	Describe methods used for assessing risk of bias of individual studies (including specification of whether this was done at the study or outcome level), and how this information is to be used in any data synthesis	6
Summary measures	13	State the principal summary measures (such as risk ratio, difference in means).	7
Synthesis of results	14	Describe the methods of handling data and combining results of studies, if done, including measures of consistency (such as I2) for each meta-analysis	7
Risk of bias across studies	15	Specify any assessment of risk of bias that may affect the cumulative evidence (such as publication bias, selective reporting within studies)	7
Additional analyses	16	Describe methods of additional analyses (such as sensitivity or subgroup analyses, meta-regression), if done, indicating which were pre-specified	7
**RESULTS**			
Study selection	17	Give numbers of studies screened, assessed for eligibility, and included in the review, with reasons for exclusions at each stage, ideally with a flow diagram	7
Study characteristics	18	For each study, present characteristics for which data were extracted (such as study size, PICOS, follow-up period) and provide the citations	8
Risk of bias within studies	19	Present data on risk of bias of each study and, if available, any outcome-level assessment.	8
Results of individual studies	20	For all outcomes considered (benefits or harms), present for each study (a) simple summary data for each intervention group and (b) effect estimates and confidence intervals, ideally with a forest plot	9
Synthesis of results	21	Present results of each meta-analysis done, including confidence intervals and measures of consistency	9
Risk of bias across studies	22	Present results of any assessment of risk of bias across studies	16

PRISMA 2009 Checklist

Section/Topic	Item No	Checklist item	Reported on page No
Additional analysis	23	Give results of additional analyses, if done (such as sensitivity or subgroup analyses, meta-regression)	16
**DISCUSSION**			
Summary of evidence	24	Summarise the main findings including the strength of evidence for each main outcome; consider their relevance to key groups (such as health care providers, users, and policy makers)	16
Limitations	25	Discuss limitations at study and outcome level (such as risk of bias), and at review level (such as incomplete retrieval of identified research, reporting bias)	20
Conclusions	26	Provide a general interpretation of the results in the context of other evidence, and implications for future research	20
FUNDING			
Funding	27	Describe sources of funding for the systematic review and other support (such as supply of data) and role of funders for the systematic review	22

From: Liberati A, Altman DG, Tetzlaff J, et al. The PRISMA statement for reporting systematic reviews and meta-analyses of studies that evaluate healthcare interventions: explanation and elaboration. *BMJ*. 2009;339:b2700. Published 2009 Jul 21. doi:10.1136/bmj.b2700.
