# Supplementary material for: Efficacy of Glutamine in Treating Severe Acute Pancreatitis: A Systematic Review and Meta-Analysis
Source: Front Nutr. 2022 Jun 14;9:865102. doi: 10.3389/fnut.2022.865102 (PMC9237617; doi:10.3389/fnut.2022.865102)
Supplement: Supplementary file 2 [file Data_Sheet_2.PDF]

Study or Subgroup	Experimental Mean	Experimental SD	Experimental Total	Control Mean	Control SD	Control Total	Weight	Mean Difference IV, Random, 95% CI
Chu 2020	30.83	4.06	42	50.02	3.27	42	0.0%	-19.19 [-20.77, -17.61]
Gao 2018	23.42	2.26	45	35.39	2.74	45	20.2%	-11.97 [-13.01, -10.93]
Sun 2019	30.68	4.14	39	49.68	3.57	39	18.8%	-19.00 [-20.72, -17.28]
Wa 2015	24.6	2.1	54	34.3	2.8	54	20.4%	-9.70 [-10.63, -8.77]
Wang 2017	23.65	2.23	49	35.13	2.71	49	20.3%	-11.48 [-12.46, -10.50]
Zhao 2017	23.6	2.2	48	35.1	2.7	48	20.3%	-11.50 [-12.49, -10.51]
**Total (95% CI)**			**235**			**235**	**100.0%**	**-12.63 [-14.89, -10.38]**

Heterogeneity: Tau² = 6.24; Chi² = 87.79, df = 4 (P < 0.00001); I² = 95%
Test for overall effect: Z = 11.00 (P < 0.00001)

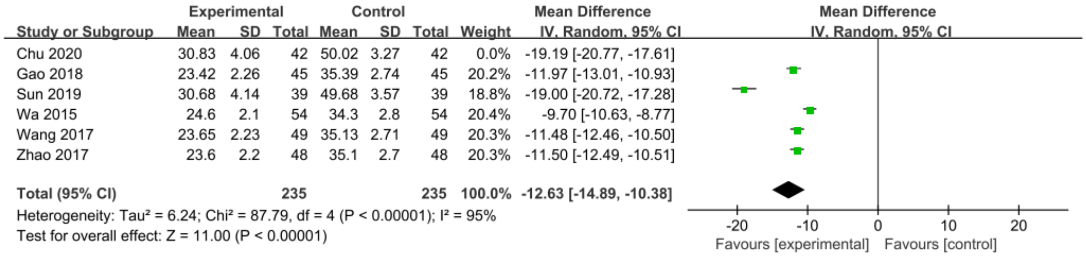

Study or Subgroup	Experimental Mean	Experimental SD	Experimental Total	Control Mean	Control SD	Control Total	Weight	Mean Difference IV, Random, 95% CI
Chu 2020	30.83	4.06	42	50.02	3.27	42	19.7%	-19.19 [-20.77, -17.61]
Gao 2018	23.42	2.26	45	35.39	2.74	45	0.0%	-11.97 [-13.01, -10.93]
Sun 2019	30.68	4.14	39	49.68	3.57	39	19.5%	-19.00 [-20.72, -17.28]
Wa 2015	24.6	2.1	54	34.3	2.8	54	20.3%	-9.70 [-10.63, -8.77]
Wang 2017	23.65	2.23	49	35.13	2.71	49	20.2%	-11.48 [-12.46, -10.50]
Zhao 2017	23.6	2.2	48	35.1	2.7	48	20.2%	-11.50 [-12.49, -10.51]
**Total (95% CI)**			**232**			**232**	**100.0%**	**-14.11 [-17.45, -10.77]**

Heterogeneity: Tau² = 14.07; Chi² = 166.81, df = 4 (P < 0.00001); I² = 98%
Test for overall effect: Z = 8.29 (P < 0.00001)

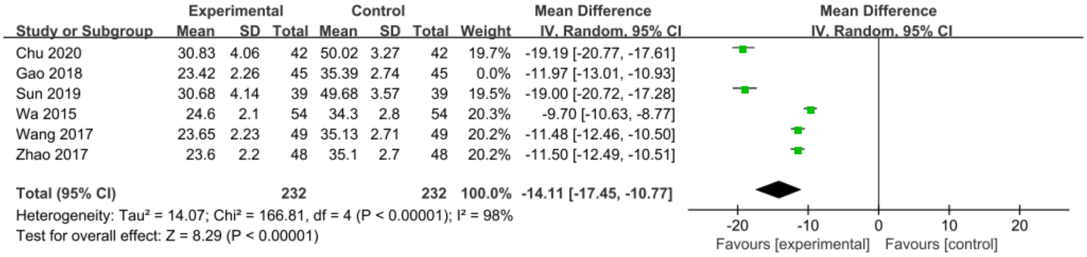

Study or Subgroup	Experimental Mean	Experimental SD	Experimental Total	Control Mean	Control SD	Control Total	Weight	Mean Difference IV, Random, 95% CI
Chu 2020	30.83	4.06	42	50.02	3.27	42	19.2%	-19.19 [-20.77, -17.61]
Gao 2018	23.42	2.26	45	35.39	2.74	45	20.1%	-11.97 [-13.01, -10.93]
Sun 2019	30.68	4.14	39	49.68	3.57	39	0.0%	-19.00 [-20.72, -17.28]
Wa 2015	24.6	2.1	54	34.3	2.8	54	20.3%	-9.70 [-10.63, -8.77]
Wang 2017	23.65	2.23	49	35.13	2.71	49	20.2%	-11.48 [-12.46, -10.50]
Zhao 2017	23.6	2.2	48	35.1	2.7	48	20.2%	-11.50 [-12.49, -10.51]
**Total (95% CI)**			**238**			**238**	**100.0%**	**-12.70 [-15.13, -10.27]**

Heterogeneity: Tau² = 7.36; Chi² = 104.70, df = 4 (P < 0.00001); I² = 96%
Test for overall effect: Z = 10.24 (P < 0.00001)

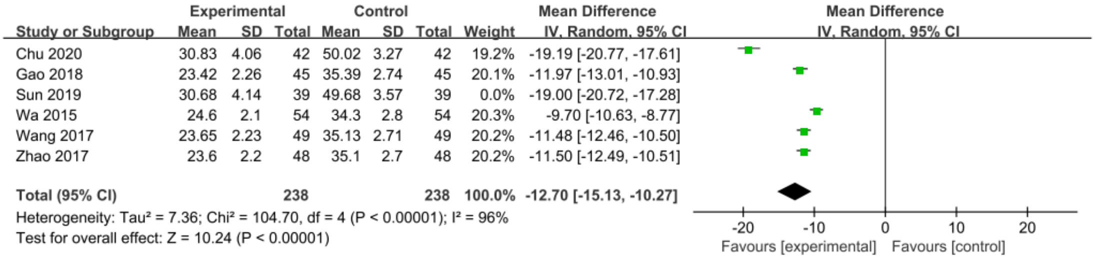

Study or Subgroup	Experimental Mean	Experimental SD	Experimental Total	Control Mean	Control SD	Control Total	Weight	Mean Difference IV, Random, 95% CI
Chu 2020	30.83	4.06	42	50.02	3.27	42	19.6%	-19.19 [-20.77, -17.61]
Gao 2018	23.42	2.26	45	35.39	2.74	45	20.3%	-11.97 [-13.01, -10.93]
Sun 2019	30.68	4.14	39	49.68	3.57	39	19.5%	-19.00 [-20.72, -17.28]
Wa 2015	24.6	2.1	54	34.3	2.8	54	0.0%	-9.70 [-10.63, -8.77]
Wang 2017	23.65	2.23	49	35.13	2.71	49	20.3%	-11.48 [-12.46, -10.50]
Zhao 2017	23.6	2.2	48	35.1	2.7	48	20.3%	-11.50 [-12.49, -10.51]
**Total (95% CI)**			**223**			**223**	**100.0%**	**-14.56 [-17.56, -11.56]**

Heterogeneity: Tau² = 11.27; Chi² = 127.86, df = 4 (P < 0.00001); I² = 97%
Test for overall effect: Z = 9.52 (P < 0.00001)

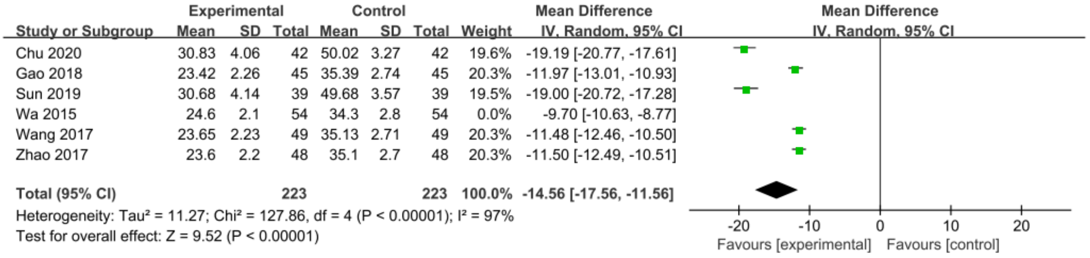

Study or Subgroup	Experimental Mean	Experimental SD	Experimental Total	Control Mean	Control SD	Control Total	Weight	Mean Difference IV, Random, 95% CI
Chu 2020	30.83	4.06	42	50.02	3.27	42	19.7%	-19.19 [-20.77, -17.61]
Gao 2018	23.42	2.26	45	35.39	2.74	45	20.2%	-11.97 [-13.01, -10.93]
Sun 2019	30.68	4.14	39	49.68	3.57	39	19.6%	-19.00 [-20.72, -17.28]
Wa 2015	24.6	2.1	54	34.3	2.8	54	20.3%	-9.70 [-10.63, -8.77]
Wang 2017	23.65	2.23	49	35.13	2.71	49	0.0%	-11.48 [-12.46, -10.50]
Zhao 2017	23.6	2.2	48	35.1	2.7	48	20.2%	-11.50 [-12.49, -10.51]
**Total (95% CI)**			**228**			**228**	**100.0%**	**-14.21 [-17.56, -10.86]**

Heterogeneity: Tau² = 14.18; Chi² = 163.80, df = 4 (P < 0.00001); I² = 98%
Test for overall effect: Z = 8.31 (P < 0.00001)

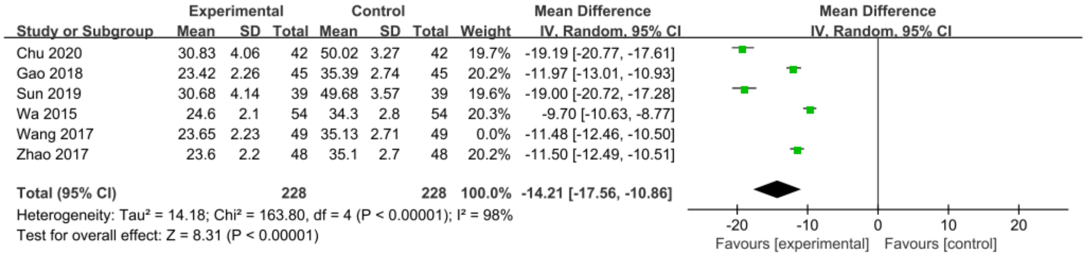

Study or Subgroup	Experimental Mean	Experimental SD	Experimental Total	Control Mean	Control SD	Control Total	Weight	Mean Difference IV, Random, 95% CI
Chu 2020	30.83	4.06	42	50.02	3.27	42	19.7%	-19.19 [-20.77, -17.61]
Gao 2018	23.42	2.26	45	35.39	2.74	45	20.2%	-11.97 [-13.01, -10.93]
Sun 2019	30.68	4.14	39	49.68	3.57	39	19.6%	-19.00 [-20.72, -17.28]
Wa 2015	24.6	2.1	54	34.3	2.8	54	20.3%	-9.70 [-10.63, -8.77]
Wang 2017	23.65	2.23	49	35.13	2.71	49	20.2%	-11.48 [-12.46, -10.50]
Zhao 2017	23.6	2.2	48	35.1	2.7	48	0.0%	-11.50 [-12.49, -10.51]
**Total (95% CI)**			**229**			**229**	**100.0%**	**-14.21 [-17.56, -10.86]**

Heterogeneity: Tau² = 14.18; Chi² = 163.99, df = 4 (P < 0.00001); I² = 98%
Test for overall effect: Z = 8.31 (P < 0.00001)

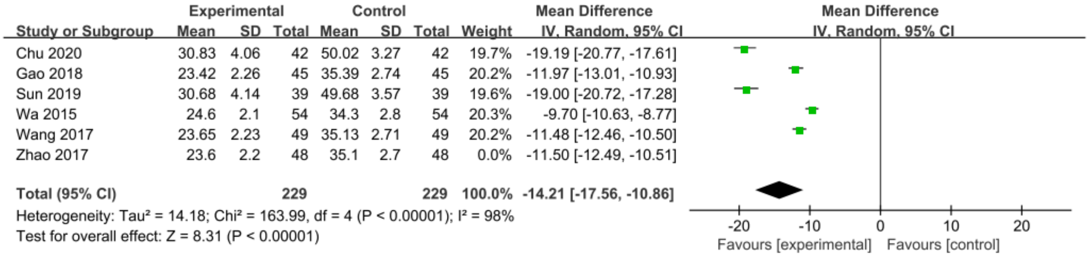

In the results of ALT, sensitivity analysis was performed by excluding individual studies

from the included studies and found that the heterogeneity was still significant. I^2 tests and *P* are the criteria for the heterogeneity test, ◆: pooled mean difference, —■—: mean difference, and the edges of ◆: 95% CI.
