# Supplementary material for: Efficacy of Glutamine in Treating Severe Acute Pancreatitis: A Systematic Review and Meta-Analysis
Source: Front Nutr. 2022 Jun 14;9:865102. doi: 10.3389/fnut.2022.865102 (PMC9237617; doi:10.3389/fnut.2022.865102)
Supplement: Supplementary file 3 [file Data_Sheet_3.PDF]

**[6] He XL, Ma QJ, Lu JG, et al.Effect of total parenteral nutrition (TPN) with and without glutamine dipeptide supplementation on outcome in severe acute pancreatitis (SAP).** ***Clinical Nutrition Supplements*****. 2004;1:43-47.**

Abstract: Objective: To investigate the effect of total parenteral nutrition (TPN) and glutamine-supplemented TPN in patients with serious acute pancreatitis (SAP). Methods: Sixty-four patients with SAP were randomly divided into three therapeutic groups. Twenty-three cases (group I) were treated by traditional therapeutic program combined with traditional therapy, the 21 cases in group II and 20 cases in group III were treated by TPN and glutamine-supplemented TPN, respectively. The concentration of serum albumin was determined, recovery time of blood amylase, the incidence of complications, mortality, length of hospital stay (LOS) and body mass of the patients in each group were monitored. Results: The concentrations of serum albumin were low in all three groups at admission. After 2 weeks of treatment, the serum albumin concentration increased in groups II and III, while it remained almost the same in group I. Mortalities for groups I, II, and III were 43.5% (10/23), 14.3% (3/21, vs. group I Po0:05) and 0% (vs. group I Po0:01), respectively. The incidence of complications in group I (21/23) was much higher than those in group II (11/21, Po0:01) and group III (4/20, Po0:01), and in group III they were less frequent than in group II (Po0:05). In fact no pancreatic infection occurred in group III with glutamine dipeptide supplementation. The loss of body mass in group I was more profound than in groups II and III, Po0:05. The length of stay (LOS) in group I (39.1710.60 d) was longer than those of group II (28.676.90 d, Po0:05) and III (25.377.60 d, Po0:01). Conclusions: Combined with conventional therapy, TPN and glutamine dipeptidesupplemented TPN therapy can reduce mortality and the incidence of infectious complications, shorten the LOS, and improve the nutritional status of patients with SAP. Glutamine-supplemented TPN therapy can apparently prevent the occurrence of pancreatic infection.

**[12] Fuentes-Orozco C, Anaya-Prado R, González-Ojeda A, et al. L-alanyl-L-glutamine-supplemented parenteral nutrition improves infectious morbidity in secondary peritonitis.** ***Clin Nutr.*** **2004;23(1):13-21.**

Abstract: Background: The effect of parenteral GLN on recovery from severe acute pancreatitis has not been thoroughly investigated. The aims of this study were to determine whether parenteral GLN improves nutrition status and immune function, and to determine its ability to reduce morbidity and mortality in patients with this condition. Methods: In a randomized clinical trial, 44 patients with severe acute pancreatitis were randomly assigned to receive either standard PN (n = 22) or L-alanyl-L-glutamine supplemented PN (n = 22) after hospital admission. Nitrogen balance, counts of leukocytes, total lymphocytes, and CD4 and CD8 subpopulations, and serum levels of immunoglobulin A, total protein, albumin, C-reactive protein, and serum interleukin (IL)-6 and IL-10 were measured on days 0, 5, and 10. Hospital stay, infectious morbidity, and mortality were also evaluated. Results: Demographics, laboratory characteristics, and pancreatitis etiology and severity at entry to the study were similar between groups. The study group exhibited significant increases in serum IL-10 levels, total lymphocyte and lymphocyte subpopulation counts, and albumin serum levels. Nitrogen balance also improved to positive levels in the study group and remained negative in the control group. Infectious morbidity was more frequent in the control group than in the study group. The duration of hospital stay was similar between groups, as was mortality. Conclusion: The results suggest that treatment of patients with GLN-

supplemented PN may decrease infectious morbidity rate compared with those who treated with nonenriched PN. (JPEN J Parenter Enteral Nutr. 2008;32:403-411).

**[25] Liu X, Sun XF, Ge QX. The role of glutamine supplemented total parenteral nutrition (TPN) in severe acute pancreatitis. *Eur Rev Med Pharmacol Sci.* 2016;20(19):4176-4180.**
Abstract: OBJECTIVE: To evaluate the role of glutamine-supplemented total parenteral nutrition (TPN) in severe acute pancreatitis. PATIENTS AND METHODS: Forty-seven patients with severe acute pancreatitis were admitted to Huaihe Hospital, China, over a period of one year (July 2013 to June 2014) were randomly divided into two therapeutic groups. Patients in group 1 (24 patients in total) and group 2 (23 patient in total) were treated with glutamine-supplemented TPN and standard TPN respectively. Patients were assessed for nutritional parameters, the prevalence of complications, mortality, length of hospital stay (LOS) and length of TPN. RESULTS: The majority of patients were male in both groups (62.5% in group 1 and 60.9% in group 2) and the average age was similar (39.13 ± 4.46 years in group 1 and 40 ± 3.96 years in group 2). The major causative factor was also similar in both groups, i.e. gall stones. The prevalence of complications in the group 2 was much higher (47.85%) than those in the group 1 (25%). The mortality rate for group 1 and 2 were 4.2% (1/24) and 17.4% (4/23), respectively. The length of hospital stay in the group 2 (23.08 ± 2.02 days) was longer than those of the group 1 (20.33 ± 2.40 days). The length of TPN was also longer in the group 2 (16.47 ± 2.72 days) than those of the group 1 (10.56 ± 2.21 days). Glutamine was also associated with significant increase in serum albumin level. CONCLUSIONS: Glutamine-supplemented TPN can reduce the mortality and the occurrence of complications, shorten the length of stay and improve the nutritional status of the patients with severe acute pancreatitis.

**[26] Chu BX. Application of glutamine combined with early enteral nutrition in patients with severe acute pancreatitis Effectiveness analysis. *World Latest Medicine Information (Electronic Version).* 2020,20(86):84,86.**
Abstract: Objective: To observe and analyze the clinical effect of glutamine (Gln) combined with early enteral nutrition (EEN) in patients with severe acute pancreatitis (SAP). Methods: A total of 84 SAP patients admitted to our hospital from October 2017 to February 2019 were selected as the research objects, and they were randomly divided into an observation group and a control group, with 42 cases in each group. Among them, the control group was given conventional treatment and EEN treatment, and the observation group was given Gln treatment on the basis of the treatment of the control group. The clinical efficacy, liver and kidney function indexes and nutritional indexes before and after treatment were compared and analyzed between the two groups. Results: The total effective rate of treatment in the observation group was 95.34%, which was significantly higher than 78.57% in the control group, and the difference was statistically significant ($P<0.05$). After treatment, the levels of various liver and kidney function indexes in the observation group were significantly lower than those in the control group, the difference was statistically significant ($P<0.05$); the nutritional index levels in the observation group were significantly higher than those in the control group, and the difference was statistically significant ($P<0.05$). Conclusion: Compared with single EEN therapy, Gln combined with EEN supportive therapy can more effectively reduce the hypercatabolism of SAP patients, inhibit inflammatory response, improve patients' nutritional status, and improve patients' immune function, with

significant clinical effects.

**[27] Cui HT, Zhao HM. Effects of glutamine combined with early enteral nutrition on bacterial shift and inflammatory response in patients with severe acute pancreatitis.** ***Modern Journal of Integrated Traditional Chinese and Western Medicine.*** **2018,27(12):1334-1337.**
Abstract: Objective: To investigate the effect of glutamine (Gln) combined with early enteral nutrition (EN) on bacterial shift and inflammatory response in patients with severe acute pancreatitis (SAP). Methods: A total of 94 SAP patients were selected as the research objects and randomly divided into an observation group and a control group. Both groups were given fasting and fasting, enzyme inhibition, acid inhibition, gastrointestinal decompression, fluid resuscitation, blood sugar control, and maintenance of water and electrolyte balance, anti-inflammatory and other treatments. Full-dose enteral nutrition was given on the first day after admission, and the observation group was supplemented with Gln on this basis, and the evaluation was performed after 7 days of treatment. Before and after treatment, the proportion of intestinal flora was compared and analyzed, and ELISA was used to detect high-sensitivity C-reactive protein (hs-CRP), interleukin-1β (IL-1β), IL-6, IL-8, IL-10, and tumor necrosis. The levels of factor-α (TNF-α), CD3+, CD4+, CD8+ levels were determined by flow cytometry, and albumin (ALB), prealbumin (PAB), transferrin (TF) level. Results: After treatment, the levels of ALB, PAB, TF, and IL-10 in the observation group were significantly higher than those in the control group (all $P < 0.05$). Serum hs-CRP, IL-1β, TNF-α, IL-6, IL- 8 levels were significantly lower than those in the control group (all $P < 0.05$); after treatment, CD3+, CD4+, CD4+ /CD8+ in the observation group were significantly higher than those in the control group and CD8 + was significantly lower than those in the control group ( $P < 0.05$); after treatment, the numbers of Bifidobacterium and Lactobacillus in the observation group were significantly higher than those in the control group, while the numbers of Staphylococcus and Escherichia coli were significantly lower than those in the control group ($P < 0.05$). Conclusion: Gln combined with early EN can significantly improve the systemic inflammatory response in SAP patients, enhance the body's immune function, prevent the shift of intestinal flora, improve nutrition metabolism, and improve the therapeutic effect. It is an ideal supportive treatment method for SAP.

**[28] Ding YD, Zhu HX. Effects of glutamine on nutritional status and immune function in patients with acute severe pancreatitis.** ***Chin J Hemorh.*** **2007(02):272-273+276.**
Abstract: Objective To investigate the effect of glutamine-intensified TPN on nutritional status and immune function in the hospital patients with acute grave pancreatitis. Methods 20 patients were divided into two groups: standard TPN group (control group),10 cases; glutamine-intensified TPN group (study group),10 cases. Period of treatment:8 days, immune function(IgG、IgA、IgM、C3、C4),serum albumin nitrogen balance were tested. Results (1)The patients' serum albumin in the study group increased significantly when compared with that of the control group ($P < 0.01$), All patients have positive nitrogen balance. (2) The patients' immune function (IgG、IgM、IgA)after TPN treatment decreased significantly, in the control group ($P<0.05$) Conclusions The treatment of glutamine-intensified TPN improved immune function and efficiency of parenteral nutrition in the hospital patients with acute grave pancreatitis.

**[29] Fan P. Effects of drugs and early enteral nutrition on severe acute pancreatitis.** ***Chinese Urban and Rural Enterprise Hygiene.*** **2021,36(05):187-188.**
Abstract: Objective: To study the clinical effect of drugs and early enteral nutrition in patients with severe acute pancreatitis. Methods: A total of 92 patients with severe acute pancreatitis who were admitted to Dagang Hospital in Binhai New Area, Tianjin from January 2015 to January 2020 were selected. According to the principle of random 1:1 grouping, the patients were divided into a combined group and a single group with 46 cases in each group. All patients were given routine treatment. On this basis, the treatment method of the combination group was glutamine combined with early enteral nutrition, and the treatment method of the single group was early enteral nutrition. Results: The acute physiology and chronic health II (APACHE II) score of the combined group was lower than that of the single group. The levels of Alb, PA, TRE and other nutritional indexes were higher than those of the single group, respectively. The first defecation, abdominal distension relief, and hospital stay were significantly lower than those of the single group. The incidence of complications, mortality and long-term mortality in the combined group were lower than those in the single group, and the differences were statistically significant ($P<0.05$). Conclusion: In the process of treating patients with severe acute pancreatitis, the addition of glutamine combined with early enteral nutrition on the basis of conventional treatment can help improve the health of patients, improve various nutritional indicators of patients, relieve and control the patient's condition. Compared with monotherapy, the effect of combination therapy is more worthy of clinical application.

**[30] Gao ZL. Clinical effect of early enteral nutrition combined with glutamine in patients with severe acute pancreatitis.** ***Journal of Clinical Medical.*** **2018,5(32):24-25.**
Abstract: Objective: To study the clinical effect of early enteral nutrition combined with glutamine in patients with severe acute pancreatitis. Methods: A total of 90 patients with severe acute pancreatitis who were treated in our hospital from December 2016 to December 2017 were selected and randomly divided into 2 groups: the control group received early enteral nutrition therapy, and the experimental group received early enteral nutrition combined with Gln treat. Results: The APACHE II score and liver function indexes of the experimental group were significantly better than those of the control group ($P<0.05$). The levels of hs-CRP, TNF-α and IL-6 in the experimental group were lower than those in the control group, but IL-10 level was higher than that of the control group, and the difference between the groups was statistically significant ($P<0.05$). Conclusion: Early enteral nutrition combined with Gln therapy in patients with severe acute pancreatitis can effectively improve the intestinal function and liver function of patients, thereby effectively relieving the patient's condition, which is worthy of popularization and application

**[31] Gu X. Application of glutamine in severe pancreatitis.** ***China Medicine Herald.*** **2008(08):65-66.**
Abstract: Objective: To observe the effect of glutamine (Gln) on nutritional status and immune function in patients with severe pancreatitis (SAP). Methods: 60 SAP patients in our hospital were randomly divided into control group and treatment group. The control group was given parenteral nutrition, and the treatment group was given parenteral nutrition plus Gln. The changes of various

indexes in the two groups were observed after treatment. Results: After treatment, the serum glutamine and serum albumin concentrations in the two groups were significantly increased (P<0.05), and the increase in the treatment group was significantly higher than that in the control group (P<0.05); the body weight was only slightly decreased (P>0.05). Conclusion: Gln can improve nutritional status, improve immune function, and reduce complications and mortality in SAP patients.

**[32] Guan YC, Zhang ZH. Effects of glutamine combined with EENs on inflammatory factors and nutritional metabolism in patients with severe acute pancreatitis. *Clinical Medical Engineering.* 2020,27(10):1359-1360.**
Abstract: Objective: To investigate the effect of glutamine combined with EENs on inflammatory factors and nutritional metabolism in patients with severe acute pancreatitis. Methods: Eighty patients with severe acute pancreatitis were randomly divided into two groups with 40 cases in each group. Both groups were given basic treatment after admission. In addition, the control group was given EENs treatment, and the observation group was given glutamine combined with EENs treatment. The inflammatory factors, nutritional metabolism indexes and complications were compared between the two groups. Results: After treatment, the levels of inflammatory factors IL-6, IL-8 and TNF-α in the two groups were significantly decreased compared with those before treatment, and the indexes in the observation group were significantly lower than those in the control group (P < 0.05). After treatment, the levels of PA in the two groups were significantly higher than those before treatment, and the levels of TRF were significantly lower than those before treatment (P < 0.05); the levels of PA and TRF in the observation group were significantly better than those in the control group (P < 0.05). The total incidence of complications in the observation group was significantly lower than that in the control group (P < 0.05). Conclusion: Glutamine combined with EENs in the treatment of patients with severe pancreatitis can reduce the level of inflammatory factors, improve nutrition metabolism, and reduce the incidence of complications.

**[33] Guo YL, Xia M. Effects of glutamine on nutritional status of patients with severe pancreatitis. *Journal of Clinical Military Medicine.* 2006(01):12-14.**
Abstract: Objective: To observe the effect of N (2)-L-alanine-L-glutamine on the nutritional status of patients with severe pancreatitis (SAP) in total parenteral nutrition. Methods: Forty-one patients with SAP were randomly divided into conventional total parenteral nutrition (TPN) group (control group) and TPN group with glutamine (glutamine group). The changes of serum albumin level, transferrin level and body weight of the two groups were observed and compared. Results: The serum albumin of the two groups at admission was (29.1±4.84) g/L in the control group and (29.3±4.50) g/L in the glutamine group, respectively. The protein levels were (29.5±4.62) and (36.8±4.57) g/L after two weeks of treatment. The body weight of the control group at admission was (61.5±14.37) kg, that of the glutamine group was (69.6±15.25) kg, and that after 2 weeks of treatment were (57.1±14.63) kg and (67.5 ± 15.92) kg. On admission, the transferrin level in the control group was (1.7±0.7) g/L, that in the glutamine group was (1.7±0.4) g/L, and the levels after 2 weeks of treatment were (1.3±0.1) g/L and (2.0±0.5) g/L. There was significant statistical difference between the glutamine group and the control group (P<0.05). Conclusion: The addition of N (2)-L-alanine-L-glutamine to the routine TPN treatment of severe pancreatitis can

significantly improve the nutritional status of patients and reduce the incidence of mortality and complications.

**[34] Huang XD, Huang M. Application of alanyl-glutamine dipeptide in severe acute pancreatitis.** ***Modern Diagnosis and Treatment.*** **2010,21(06):342-343.**
Abstract: Objective: To observe the effect of alanyl-glutamine dipeptide-enhanced total parenteral nutrition on nutritional status and immune function in patients with severe acute pancreatitis (SAP). Methods: 48 SAP patients were randomly divided into the control group and the treatment group. On the basis of conventional treatment, the two groups were all given equal calorie and equal nitrogen doses of parenteral nutrition, but the treatment group was given glutamine dipeptide in the parenteral nutrition. The changes of nutritional status, immune function and APACHE II score in the two groups after treatment were observed. Results: After treatment, the serum albumin and immunoglobulin concentrations in the two groups were increased, but the increase in the treatment group was significantly higher than that in the control group ($P<0.05$). And the C-reactive protein (CRP) concentration and APCHE II score in the treatment group were significantly lower than those in the control group ($P<0.05$). Conclusion: Alanyl-glutamine dipeptide-enhanced total parenteral nutrition can improve the nutritional status of SAP patients, improve the immune function, reduce the APACHE II score and improve the prognosis.

**[35] Jin SL, Li LP. Efficacy of early enteral compound glutamine in the treatment of severe pancreatitis.** ***Chinese Journal of Practical Medicine.*** **2014,41(11):122-123.**
Abstract: Objective: To observe the effect of compound glutamine enteric-coated capsules in enteral nutrition solution on acute severe pancreatitis. Methods: A total of 49 patients with acute severe pancreatitis were randomly divided into 26 cases in the treatment group and 23 cases in the control group. Both groups were given conventional western medicine treatment, and enteral nutrition was started on the third day of treatment, while the treatment group was given compound glutamine. The efficacy and adverse reactions of the two groups were compared. Results: The intestinal function and APACHE II score of the treatment group were better than those of the control group, the infection rate and the length of hospital stay were lower than those of the control group ($P < 0.05$), and there was no significant difference in adverse reactions ($P > 0.05$). Conclusion: Early enteral administration of compound glutamine can improve the curative effect and improve the prognosis of acute severe pancreatitis.

**[36] Lei W, Wang YL. Glutamine combined with early jejunal nutrition in the treatment of severe acute pancreatitis clinical research.** ***Laboratory Medicine and Clinical.*** **2016,13(06):819-822.**
Abstract: Objective: To preliminarily explore the clinical efficacy of glutamine combined with early jejunal nutrition (EENs) in the treatment of severe acute pancreatitis (SAP). Methods: A total of 76 SAP patients admitted to our hospital from January 2012 to June 2014 were randomly divided into the study group and the control group, 38 cases in each group. The control group was treated with EENs, and the study group was treated with glutamine combined with early jejunal nutrition (GEENs). The levels of tumor necrosis factor-α (TNF-α), interleukin-6 (IL-6), high-sensitivity C-reactive protein (hs-CRP), peripheral serum levels on the 1st, 3rd, 7th, and 14th days after admission were compared between the two groups of patients. The levels of immunoglobulin

A (IgA), immunoglobulin M (IgM), and immunoglobulin G (IgG) were recorded, and the APACHE II score was recorded at the same time, and the abdominal pain relief, abdominal signs disappeared, and gastrointestinal function returned to normal time and average days of hospitalization, total effective rate, case fatality rate and complication rate in the two groups were observed in the two groups of patients. Results: Abdominal pain relief, abdominal signs disappeared, and gastrointestinal function returned to normal time in the study group were significantly lower than those in the control group. There was no significant difference in the average length of hospital stay between the two groups (P>0.05), and there was no significant difference in the incidence of complications and mortality between the two groups (P>0.05). There was no significant difference in the APCHE II score between the two groups of patients before admission (P>0.05). The scores of the study group were significantly lower than those of the control group at 7 and 14 days of treatment, and the difference between the two groups was statistically significant (P<0.05). On the third day of treatment, the hs-CRP level of the study group was significantly lower than that of the control group, and the difference was statistically significant (P<0.001). On the 7th day, the level of IL-6 was significantly lower than that of the control group, and the difference was statistically significant (P<0.05). The level of TNF-α was significantly lower than that of the control group on the 14th day, and the difference was statistically significant (P<0.01). Compared with the control group, the IgG level of the patients in the study group was significantly increased on the seventh and 14th days, and the difference was statistically significant (P<0.05; P<0.01), but There was no significant difference in IgA and IgM levels between the two groups of patients during treatment (P>0.05). Conclusion: GEENs can effectively improve the general clinical conditions of SAP patients by regulating the inflammatory response and immune function in SAP patients, but they cannot Shorten hospital stay.

**[37] Ran J, Zhu Y. Effect of glutamine on disease course in severe pancreatitis patients. *World Chinese Journal of Digestology.* 2014,22(33):5159-5163.**

Abstract: AIM: To assess the influence of different parenteral nutrition (PN) regimens on the course of disease in severe acute pancreatitis (SAP) patients. METHODS: Clinical data for 50 SAP patients treated at our hospital from January 2010 to June 2013 were retrospectively analyzed. The patients were randomly divided into either a study group (n = 25) or a control group (n = 25). Both groups underwent conventional PN therapy for one week. The study group was additionally given glutamine. The levels of amylase in urine and blood, liver function, renal function, routine blood parameters, serum complement, APACHE II score, Balthazar CT score, total hospital stay, ICU stay, mechanical ventilation time, time to recovery of intestinal function and the incidence of complications were compared between the two groups. RESULTS: After treatment, the levels of amylase in urine and blood and renal function showed a declining trend, and there were no statistically significant differences between the two groups (P > 0.05). Alanine aminotransferase (46.3 U/L ± 30.1 U/L vs 25.1 U/L ± 21.3 U/ L), aspartase aminotransferase (31.0 U/L ± 15.3 U/L vs 20.5 U/L ± 11.7 U/L), white blood cell count (14.8 × 109 /L ± 4.3 × 109 /L vs 8.7 × 109 /L ± 2.1×109 /L) and C-reactive protein (39.8 mg/L ± 6.5 mg/L vs 30.2 mg/L ± 6.3 mg/L) were significantly higher in the control group than in the study group (P < 0.05). APACHE II score and Balthazar CT score had no significant differences between the two groups (P > 0.05). The levels of IgA (2.4 g/L ± 0.2 g/L vs 1.7 g/L ± 0.2 g/L), IgG (9.8 g/L ± 1.2 g/L vs 8.7 g/L ± 1.0 g/L) and IgM (1.2 g/L ± 0.9 g/L vs 0.7 g/L ± 0.8 g/L) were significantly higher in the study group

($P < 0.05$). Total hospital stay (32.3 d $\pm$ 6.2 d vs 20.6 d $\pm$ 4.5 d), ICU stay (13.9 d $\pm$ 3.1 d vs 10.2 d $\pm$ 2.5 d), mechanical ventilation time (8.4 d $\pm$ 2.1 d vs 5.2 d $\pm$ 1.3 d) and the time to recovery of intestinal function (4.4 d $\pm$ 1.2 d vs 3.6 d $\pm$ 1.0 d) were all significantly higher in the control group ($P < 0.05$). CONCLUSION: Gln containing PN regimen could significantly shorten hospital stay and mechanical ventilation time, reduce the risk of infection and other complications, and improve prognosis in SAP patients

**[38] Ren WS, Guo YL, Zhang T. Clinical efficacy analysis of glutamine combined with early enteral nutrition in the treatment of severe acute pancreatitis. *Guizhou Medicine.* 2019,43(12):1887-1889.**

Abstract: Objective: To observe the clinical efficacy and safety of glutamine combined with early enteral nutrition in the treatment of severe acute pancreatitis, and to provide reference for the selection of clinical treatment options for acute severe pancreatitis. Methods: A total of 60 patients with severe acute pancreatitis who were treated in our hospital from January 2016 to January 2019 were selected, numbered according to the order of enrollment, and randomly divided into a control group and a study group by numerical random table method, with 30 cases in each group. Both groups were given routine treatment for acute pancreatitis, the control group was additionally given early enteral nutrition therapy, and the study group was additionally given early enteral nutrition combined with glutamine therapy. Both groups were treated for 2 weeks as a course of treatment. The clinical symptoms and signs (abdominal pain improvement time, blood AMY recovery time) improvement time, inflammatory factors (IL-6, IL-10, CRP, TNF-α) and APACHE II scores before and after treatment were observed in the two groups. The prognostic indicators (conversion rate, serious complication rate, and mortality rate) of the two groups of patients were compared, and the drug non-response rate during treatment was compared between the two groups. Results: (1) Abdominal pain improvement time and blood AMY recovery time in the study group were shorter than those in the control group ($P<0.05$); (2) There was no significant difference in IL-6, IL-10, CRP and TNF-α between the two groups when they were enrolled ($P>0.05$). After completing one course of treatment, the scores of IL-6, CRP, and TNF-α in the two groups were significantly decreased, and the research group was lower than the control group, IL-10 was increased compared with before treatment, and the research group was higher than the control group, The difference was statistically significant ($P<0.05$); (3) There was no significant difference in the APACHE II score between the two groups at the time of enrollment ($P>0.05$). After completing one course of treatment, the APACHE II scores of the two groups were decreased compared with those before treatment, and the research group was lower than the control group ($P<0.05$); (4) The conversion rate and serious complication rate of the research group were lower than those of the control group. The difference was statistically significant ($P<0.05$); (5) there was no significant difference in the adverse drug reaction rate between the two groups ($P>0.05$). Conclusion: Enteral nutrition combined with glutamine in the treatment of severe acute pancreatitis can effectively improve the short-term efficacy and prognosis of patients without increasing the adverse drug reaction rate of patients, which has high clinical value.

**[39] Sun YB. Effects of glutamine and early enteral nutrition on patients with severe acute pancreatitis. *Practical Clinical Journal of Integrated Traditional Chinese and Western Medicine.* 2019,19(09):17-19.**

Abstract: Objective: To investigate the effect of glutamine and early enteral nutrition on the curative effect and liver and kidney function of patients with severe acute pancreatitis (SAP). Methods: A total of 78 SAP patients who were treated from September 2016 to March 2019 were selected as the research objects, and divided into the control group and the observation group by random number table, 39 cases in each group. The control group was given conventional treatment and early enteral nutrition treatment. The observation group was treated with glutamine on the basis of the control group. The curative effect, liver and kidney function and nutritional indexes were compared between the two groups before and after. Results: After treatment, the total effective rate of the observation group was higher than that of the control group, the difference was statistically significant, P<0.05. The levels of alanine aminotransferase, aspartate aminotransferase, blood urea nitrogen and serum creatinine in the observation group were lower than those in the control group, and the difference was statistically significant, P<0.05; The levels of nutritional indexes such as albumin and transferrin were higher than those in the control group, and the difference was statistically significant, P<0.05. Conclusion: The application of glutamine and early enteral nutrition in the treatment of SAP can improve the liver and kidney function and nutritional metabolism, and improve the therapeutic effect.

**[40] Tong ZH, Li WQ, Yu WK, et al. Effects of early enteral nutrition supplementation with glutamine on inflammatory response and immune function in severe acute pancreatitis. *Parenteral & Enteral Nutrition.* 2009,16(05):264-268.**

Abstract: Objective: To observe the effect of adding Gln to EEN on inflammatory response and immune function in severe acute pancreatitis (SAP). Methods: 60 patients were randomly divided into three groups. The conventional EN group received EN on the 7th to 10th day of admission; the EEN group received EN on the 2nd to 4th day; the Gln enhanced group received Gln 0.6g/(kg· d). The APACHE II score was performed and the CRP, IL-6, Gln concentrations and monocyte surface human leukocyte antigen (HLA-DR) were detected at the time of admission, 2, 5, 7, 10, 14 and 30 days after admission; recorded before and after EN The patient's daily bladder pressure and bowel sounds. When the bladder pressure > 25cmH2O and bowel sounds disappeared, EN was discontinued. Results: ① The routine EN group, EEN group and Gln group completed clinical observation in 17 cases, 16 cases and 16 cases respectively. The start time of EN in the routine EN group was later than that in the EEN group and the Gln group (P<0.05), but there was no significant difference between the three groups in the comparison of tolerance, time to full dose, duration, EN support pathway and complications (P>0.05). ②In the early stage of the disease, the APACHE II score, CRP and IL-6 of the patients in the routine EN group were higher than those in the EEN group and the Gln group, and the concentrations of HLA-DR and Gln were lower (P<0.05). The IL-6 in the Gln group was lower than that in the EEN group. However, the concentrations of HLA-DR and Gln were higher (P<0.05); ③ The APCHE II score, the incidence of multiple organ dysfunction syndrome (MODS) and bladder pressure before EN were significantly higher in those who had not completed the clinical observation than those who had completed the clinical observation. (P<0.05). Conclusion: The early addition of Gln to EN solution in SAP patients has more obvious effects on increasing blood Gln concentration, reducing early inflammatory response and improving immune function.

**[41] Wa YL. Curative effect of early enteral compound glutamine in treatment of severe**

**pancreatitis.** ***World Chinese Journal of Digestology.*** **2015,23(9): 1484-1488.**
Abstract: AIM: To assess the curative effect of early enteral compound glutamine application in patients with severe acute pancreatitis (SAP). METHODS: One hundred and eight SAP patients who were treated from January 2012 to January 2014 at Shanghai Red Cross Hospital were randomly divided into either an observation group (n = 54) or a control group (n = 54). The control group was given early enteral nutrition, and the observation group was given early enteral nutrition combined with compound glutamine. Curative effects, liver and kidney function, acute physiology and chronic health evaluation II (APACHE II) score, serum tumor necrosis factor alpha (TNF-α), high-sensitivity C-reactive protein (hs-CRP), interleukin 1B (IL-1B), IL-8, and IL-6 were compared for the two groups. RESULTS: APACHE II score, alanine transaminase (ALT), blood urea nitrogen (BUN), total bilirubin (TBIL), aspartate transaminase (A S T) a n d S c r b e f o r e t r e a t m e n t w e r e significantly higher than those after treatment in both groups. After treatment, APACHE II score, ALT, BUN, TBIL, AST and Scr were significantly lower in the observation group compared with the control group ($P < 0.05$). After treatment, serum levels of TNF-α, hs-CRP, IL-1B, IL-8, and IL-6 were significantly decreased and IL-10 was significantly elevated in both groups ($P < 0.05$). After treatment, serum TNF-α, hs-CRP, IL-1B, IL-8, and IL-6 were significantly lower and IL-10 was significantly higher in the observation group than in the control group ($P < 0.05$). CONCLUSION: Early enteral compound glutamine is able to significantly improve systemic inflammatory state and has better clinical curative effect in patients with SAP.

**[42] Wang LF. Glutamine combined with early jejunal nutrition in the treatment of 98 cases of severe acute pancreatitis.** ***Medical Journal of Chinese People's Health.*** **2017,29(19):36-37.**
Abstract: Objective: To observe the application effect of glutamine combined with early jejunal nutrition (GEENs) in patients with severe acute pancreatitis (SAP). Methods: A total of 98 SAP patients were selected as the observation objects, and the patients were divided into the control group and the study group with 49 cases in each group by computer random allocation. The control group was given early jejunal nutrition, and the study group was treated with glutamine on the basis of the control group, and the levels of liver function indexes and inflammatory cytokines were compared between the two groups before and after treatment. Results: The liver function indexes in the study group were significantly better than those in the control group, and the levels of various inflammatory cytokines were significantly lower than those in the control group, with statistical significance ($P<0.05$). Conclusion: GEENs treatment can significantly improve the liver function of SAP patients, reduce the systemic inflammatory response of the patients, help to improve the clinical symptoms of the patients, and promote the recovery.

**[43] Wang S, Huang SW, Liu P, et al. Significance of total parenteral nutrition combined with glutamine dipeptide in non-surgical treatment of patients with severe pancreatitis.** ***Practical Preventive Medicine.*** **2009,16(03):844-846.**
Abstract: Objective: To observe the effect of total parenteral nutrition combined with glutamine dipeptide in the non-surgical treatment of acute severe pancreatitis. Methods: 48 patients with acute severe pancreatitis were randomly divided into two groups. Group I was treated with total parenteral nutrition (TPN) on the basis of traditional conservative treatment principles, and group II was treated with glutamine dipeptide based on the regimen of group I. Serum albumin level,

plasma glutamine concentration, nitrogen balance, T lymphocyte transformation rate and other indicators were determined. Results: After 2 weeks of treatment, the serum albumin, plasma glutamine concentration, nitrogen balance and T lymphocyte transformation rate in group II were higher than those in group I ($P<0.05$). Conclusion: Glutamine dipeptide combined with TPN therapy on the basis of traditional non-surgical treatment of acute severe pancreatitis can significantly improve the nutritional status of patients, promote the recovery of nitrogen balance, improve immunity, reduce mortality and the incidence of complications.

**[44] Wu D, Li Y. Influence of early enteral administration of glutamine on clinical prognosis in patients with severe acute pancreatitis. *Journal of Jiujiang University (Natural Science Edition).* 2010,25(01):110-111+116.**

Abstract: Objective: To observe the curative effect of early immunoenteral nutrition (EN) through nasojejunal tube in the treatment of acute severe pancreatitis (SAP). Methods: 30 patients with SAP were selected, and 15 cases of SAP were treated with glutamine (Gln) in the early stage through nasojejunal tube (treatment group, EEN1 group), and 15 cases of SAP were treated with standard enteral nutrition through nasojejunal tube in the early stage (control group EEN2 group). The treatment results were compared to observe the differences in serum albumin, serum prealbumin, complication rate, mortality rate, hospitalization time and average hospitalization cost. Results: The early implementation of immune enteral nutrition through nasojejunal tube can significantly reduce the incidence of complications and reduce the mortality rate. There was no statistical difference in hospitalization time and hospitalization costs. Conclusion: The rational application of EN, especially the early enteral glutamine immunoenteral nutrition, can significantly improve the clinical prognosis of patients with acute severe pancreatitis.

**[45] Yang C, Wen J, Xia M, et al. Glutamine nutritional support on intestinal mucosal barrier function and inflammatory response in patients with acute severe pancreatitis degree of influence. *Journal of Hainan Medical College*. 2017,23(14):18961899.**

Abstract: Objective: To study the effect of glutamine nutritional support on the intestinal mucosal barrier function and the degree of inflammatory response in patients with acute severe pancreatitis. Methods: Patients with severe acute pancreatitis who were treated in our hospital were selected as the research subjects and randomly divided into two groups. The patients in the control group received conventional symptomatic treatment and conventional enteral nutrition intervention, while the Gln group received conventional symptomatic treatment and glutamine enteral nutrition. intervention. Before and after treatment, the contents of intestinal mucosal barrier damage marker molecules and inflammatory mediators in serum and the expression of inflammatory signaling molecules in peripheral blood were detected. After treatment, the amount of intestinal flora was measured. Results: After treatment, the levels of LPS, DAO, HBD2, TNF-α, sTREM-1, IL-1β and IL-6 in serum of the two groups of patients and the mRNA levels of TLR4, NF-kB, MyD88 and p38MAPK in peripheral blood mononuclear cells were significantly increased. Serum LPS, DAO, HBD2, TNF-α, sTREM-1, IL-1β and IL-6 contents in Gln group after treatment as well as TLR4, NF-kB, MyD88 and p38MAPK mRNA expression in peripheral blood mononuclear cells were significantly lower than the control group. The numbers of Lactobacillus, Bifidobacterium, and Bacteroides were significantly higher than those in the control group, and the numbers of Escherichia coli and Enterococcus were significantly lower than those in the control group.

Conclusion: Glutamine nutritional support for acute severe pancreatitis can reduce the damage of intestinal mucosal barrier function and inhibit the activation of inflammatory response.

**[46] Yang TY, Zhang XY, Jiang MX. Clinical study of early enteral immunonutrition in severe acute pancreatitis.** ***Clinical Medicine of China.*** **2013,29(09):922-925.**

Abstract: Objective: To compare the efficacy and clinical value of early enteral immunonutrition (EIN) with glutamine and early standard formula enteral nutrition (EEN) in patients with severe acute pancreatitis (SAP). Methods: 28 SAP patients were selected and divided into two groups by random number table. After admission, they were given fasting, anti-infection, gastrointestinal decompression, acid suppression, spasmolysis, pancreatic enzyme suppression, insulin use to reduce pancreatic burden, water and electrolyte balance maintenance and parenteral nutrition and other treatments. 72 hours after admission, the patients were placed with a spiral nasojejunal tube. The EEN group was given Bapu's enteral nutrition preparation, while the EIN group was given a small amount of warm water to dissolve glutamine granules and then added to the Bapu's enteral nutrition preparation, with a total amount of 0.2 g/(kg·d), divided into 3 times a day, and gradually reduce the dose until the patient completely returns to normal diet until it stops. The changes of APACHE II and Ranson scores, as well as the changes of inflammatory indexes, nutritional indexes and immune indexes of the two groups of patients were observed, and the time of first defecation, ICU monitoring time, total hospitalization time, total hospitalization expenses, complication rate and fatality rate were counted. Results: Both groups tolerated early enteral nutrition support therapy. The immune index IgG (19.14±2.03) in the EIN group was significantly higher than that in the EEN group (13.79±3.29) on the 14th day of treatment. Statistical significance ($P<0.05$). The time of first defecation, ICU monitoring time and total hospitalization expenses of patients in the EIN group ((4.29±1.64), (2.57±0.85), (34.36±3.59)) were significantly lower than those in the EEN group ((5.36±0.84), (3.64±1.60), and (43.86±9.43)), the differences were statistically significant (t values were 2.179, 2.213, 3.518, both $P<0.05$). Conclusion: It is safe and feasible to implement EIN in SAP patients. Both EIN and EEN can significantly reduce the inflammatory response and improve nutritional status of SAP patients, but EIN is better than EEN in improving the immune function and prognosis of SAP patients.

**[47] Yang WH, Wan X, Dai WC, et al. Efficacy analysis of glutamine combined with early enteral nutrition in the treatment of severe acute pancreatitis.** ***Smart Healthcare.*** **2021,7(22):100-102.**

Abstract: Objective: To investigate the effect of Glutamine Combined with early enteral nutrition in the treatment of severe acute pancreatitis. Methods: 65 patients with severe acute pancreatitis in the Second Affiliated Hospital of Zhengzhou University from September 2017 to September 2020 were selected and randomly divided into control group (32 cases) and glutamine group (33 cases). The control group was treated with early enteral nutrition, and the glutamine group was treated with glutamine on the basis of the control group. Results: The total effective rate of glutamine group (93.94%, 31/33) was significantly higher than that of control group (71.88%, 23/32), the difference was statistically significant ($P < 0.05$). Conclusion: On the basis of early enteral nutrition treatment, combined with glutamine treatment, can effectively improve the treatment effect, improve the nutritional status and critical degree of patients, and the clinical application effect is reliable.

**[48] Yin HY. The value of TPN and alanyl-glutamine dipeptide in the non-surgical treatment of severe acute pancreatitis. *Journal of Clinical Medical.* 2016,3(38):7521-7522.**

Abstract: Objective: To explore the clinical application value of TPN, alanyl-glutamine dipeptide in the non-surgical treatment of severe acute pancreatitis. Methods: A total of 60 patients with acute severe cholangitis treated in our hospital from August 2014 to August 2015 were selected as the research subjects, and they were randomly divided into three groups, A, B, and C, with 20 patients in each group. Among them, patients in group A were given routine conservative treatment, patients in group B were given routine conservative treatment combined with total parenteral nutrition (TPN) therapy; patients in group C were given routine conservative treatment, total parenteral nutrition therapy and comprehensive treatment method of alanyl-glutamine dipeptide was used to compare and analyze the clinical treatment effect of the three groups of patients. Results: According to statistics, there was no significant difference in serum albumin levels among the three groups before treatment ($P>0.05$). After treatment, the patients in group A were (30.2±4.61) g/L and those in group B were (36.1 ±4.53) g/L, and group C was (37.1±4.47) g/L. Compared with before treatment, the serum albumin levels of group B and group C were significantly higher, and the difference was statistically significant ($P<0.05$). At the same time, the body weight level of patients in group B and group C was significantly lower than that in group A, and the difference was statistically significant ($P<0.05$). In addition, the mortality and complication rates of patients in groups A, B, and C were significantly different ($P<0.05$). The blood starch recovery time, abdominal distension relief time and total hospitalization time of patients in group B and group C were significantly better than those in group A, and the difference was statistically significant ($P<0.05$). Conclusion: TPN, alanyl-glutamine dipeptide has a very prominent clinical therapeutic effect in the non-surgical treatment of severe acute pancreatitis. After treatment, the patient's condition has been significantly improved, and the incidence of clinical complications has been significantly improved. It is worthy of widespread clinical application.

**[49] Yuan XX, Zan JB, Xu AZ, et al. Effects of early enteral nutrition combined with Gln on systemic inflammatory response and immune function in patients with SAP. *Journal of Hepatopancreatobiliary Surgery.* 2018,30(01):22-25.**

Abstract: Objective: To evaluate the effect of early enteral nutrition (EEN) supplemented with glutamine (Gln) for patients with severe acute pancreatitis (SAP) on systemic inflammatory response and immune function. Methods: Forty-nine SAP patients admitted to the General Surgery of Anqing Hospital Affiliated to Anhui Medical University from Oct. 2015 to May. 2017 were randomly divided into the experimental group (24 cases) and the control group (25 cases). EEN combined with Gln was used in the experimental group and only EEN was used in the control group. Blood samples from all the patients were collected for analysis of inflammatory markers [C-Reactive protein (CRP), procalcitonin (PCT), Interleukin-6 (IL-6), Interleukin-8 (IL-8), tumor necrosis factor-α (TNF-α)] and immune indices (CD3+, CD4+, CD4+/CD8+) on the day l, 6 and 11 after admission, and their differences were compared between the two groups. Results: (1) On the first day after admission, compared with the control group, there was no significant difference of systemic inflammation markers (including CRP, PCT, IL-6, IL-8, TNF-α) and immune parameters (CD3+, CD4+, CD4+/CD8+) in the experimental group. (2) On day 6, the levels of

inflammatory markers CRP [(56.31±18.04) mg/L vs (98.12±11.54) mg/L, P=0.037], IL-6 [(45.87±10.99) pg/mL vs (54.72±4.77) mg/L, P=0.026), IL-8 [(45.15±9.27) ng/mL vs (59.03±4.87) ng/mL, P=0.013], TNF-α [(24.35±7.15) pg/mL vs (32.41±4.22) pg/mL, P=0.010] and PCT [(1.11±0.49) ng/mL vs (1.50±0.30) mg/L, P=0.024] in the experimental group were significantly lower than those in the control group. In addition, levels of immune indices CD3+ [(63.42±2.86) % vs (54.67±1.76) %, P=0.035], CD4+ [(43.69±1.83) % vs (40.08±1.08) %, P=0.014] and CD4+/CD8+ [(2.25±0.39) vs (1.87±0.22), P=0.006] were significantly higher in the experimental group. (3) On day 11, the levels of CRP [(16.81±7.56) mg/L vs (43.01±2.74) mg/L, P=0.009], IL-6 [(29.39±7.92) pg/mL vs (38.29±5.33) pg/mL, P=0.020], IL-8 [(30.73±9.26) ng/mL vs (39.69±5.26) ng/mL, P=0.018], TNF-α [(13.87±5.67) pg/mL vs (20.35±3.82) pg/mL, P=0.019] and PCT [(0.34±0.11) ng/mL vs (0.95± 0.29) ng/mL, P=0.035] in the experimental group were also significantly lower than those in the control group. And the levels of CD3+ [(69.68±1.87) % vs (61.14±1.21) %, P=0.017], CD4+ [(54.27±3.23) % vs (45.30±1.59) %, P=0.007] and CD4+/CD8+ [(2.98±0.51) vs (2.01±0.28), P=0.012] in the experimental group were also significantly higher. Conclusion: For patients with severe acute pancreatitis, EEN combined with Gln is superior to EEN alone in reducing systemic inflammatory response and improving immune function.

**[50] Zhao CJ, Chen X, Chen J, et al. Application of early enteral nutrition combined with glutamine in severe acute pancreatitis.** ***China Continuing Medical Education.*** **2017,9(05):135-137.**

Abstract: Objective: To assess the curative effect of early enteral nutrition combined with glutamine application in patients with severe acute pancreatitis (SAP). Methods: 96 cases of SAP patients who received clinical research in Inner Mongolia Baogang Hospital from January 2014 to June 2016 were chosen. Randomly 96 patients were divided into observation group and control group, 48 cases in each. The early enteral nutrition (EN) was given to the control group, and the observation group was given early enteral nutrition combined with compound glutamine. Curative effects, acute physiology and chronic health evaluation II (APACHE II) score, liver and kidney function, setum albumin, serum tumor necrosis factor alpha (TNF-α), high-sensitivity C-reactive protein (hs-CRP), interleukin 6 (IL-6) and IL-10 were compared for the two groups. Results: On the seventh day after treatment, APACHE II score, ALT, TBIL, BUN were significantly lower in the observation group compared with the control group (P < 0.05), ALB were significantly higher in the observation group compared with the control group (P < 0.05). Serum levels of TNF-α, hs-CRP, and IL-6 were lower and IL-10 was higher in the observation group than in the control group (P < 0.05). Conclusion: Early enteral nutrition combined with glutamine is able to significantly improve Clinical symptoms and inhibiting inflammatory response in patients with SAP.

**[51] Singh N, Mishra SK, Sachdev V, et al. Effect of oral glutamine supplementation on gut permeability and endotoxemia in patients with severe acute pancreatitis: a randomized controlled trial. Pancreas. 2014;43(6):867-873.**

Abstract: Objective: The aim of this study is to evaluate the effect of oral glutamine (GL) supplementation on gut permeability and endotoxemia (surrogate end point) in patients with severe acute pancreatitis. Methods: In a randomized controlled trial, patients were randomized to be given placebo or GL for 7 days. The primary outcome measures include the effect on gut

permeability (assessed by lactulose/mannitol excretion in urine and endotoxemia assessed by endotoxin core antibodies type IgG and IgM (EndoCab IgG and IgM). The secondary outcome measures include infectious complications, mortality, total hospital/intensive care unit stay, C-reactive protein, and prealbumin levels. Results: Patients were assigned to GL (n = 41) and placebo (n = 39) groups. There was no change in gut permeability after the intervention. However, the EndoCab IgM levels increased significantly (33 [4, 175] to 40 [8, 350] GMU/mL; P = 0.0164) and the C-reactive protein levels decreased significantly (133 [1, 287] to 88 [1, 267] ng/mL; P = 0.0236) in the GL group. No difference was observed in infectious complication, prealbumin value, hospital/intensive care unit stay, and mortality in both groups. Conclusions: No significant trend was identified for an effect of GL on gut permeability. Decreased inflammation and endotoxemia did not translate into reduced infectious complications in severe acute pancreatitis. However, the study was underpowered to detect the aforementioned difference.

**[52] Arutla M, Raghunath M, Deepika G, et al. Efficacy of enteral glutamine supplementation in patients with severe and predicted severe acute pancreatitis- A randomized controlled trial. Indian J Gastroenterol. 2019;38(4):338-347.**

Abstract:

Background: In severe acute pancreatitis (AP), intravenous glutamine has been shown to reduce the rate of complications, hospital stay, and mortality. In the present randomized trial, we aimed to evaluate the effect of enteral glutamine supplementation on clinical outcomes, gut permeability, systemic inflammation, oxidative stress, and plasma glutamine levels in patients with severe and predicted severe AP. Methods: Patients with AP admitted within 72 h of onset of symptoms were included. The primary outcome measure was development of infected pancreatic and peri-pancreatic necrosis and in-hospital mortality. High-sensitivity C-reactive protein (HS-CRP) and interleukin-6 (IL-6) were evaluated as markers of inflammation; plasma thiobarbituric acid reactive substances (TBARS) and activities of serum superoxide dismutase and glutathione peroxidase were determined to evaluate oxidative stress; serum polyethylene glycol (PEG) was tested for intestinal permeability; subjective global assessment (SGA) was used for nutritional assessment, and an improvement in organ function was measured by the Modified Marshall score. Intention-to-treat analysis was used. A $p$-value of $< 0.05$ was considered statistically significant. Results: After power calculation, we enrolled 18 patients in the glutamine and 22 in the control arm. There was no significant improvement in the development of infected necrosis and in-hospital mortality between the groups. Improvement in Modified Marshall score was observed in a higher proportion of patients receiving glutamine (15 [83.3%] vs. 12 [54.5%]; $p = 0.05$). Plasma glutamine levels improved more in glutamine-treated group (432.72 ± 307.83 vs. 618.06 ± 543.29 μM/L; $p = 0.004$), while it was lower in controls (576.90 ± 477.97 vs. 528.20 ± 410.45 μM/L; $p = 0.003$). PEG level was lower after glutamine supplementation (39.91 ± 11.97 vs. 32.30 ± 7.39 ng/mL; $p = 0.02$). Statistically significant reduction in IL-6 concentration was observed in the glutamine group at the end of treatment (87.44 ± 7.1 vs. 63.42 ± 33.7 μM/L; $p = 0.02$). Conclusion: Despite absence of improvement in infected necrosis and in-hospital mortality, enteral glutamine supplementation showed improvement in gut permeability, oxidative stress, and a trend towards improvement in organ function as depicted by improvement in the Modified Marshall score.
