# Supplementary material for: Efficacy of Glutamine in Treating Severe Acute Pancreatitis: A Systematic Review and Meta-Analysis
Source: Front Nutr. 2022 Jun 14;9:865102. doi: 10.3389/fnut.2022.865102 (PMC9237617; doi:10.3389/fnut.2022.865102)
Supplement: Supplementary file 4 [file Data_Sheet_4.PDF]

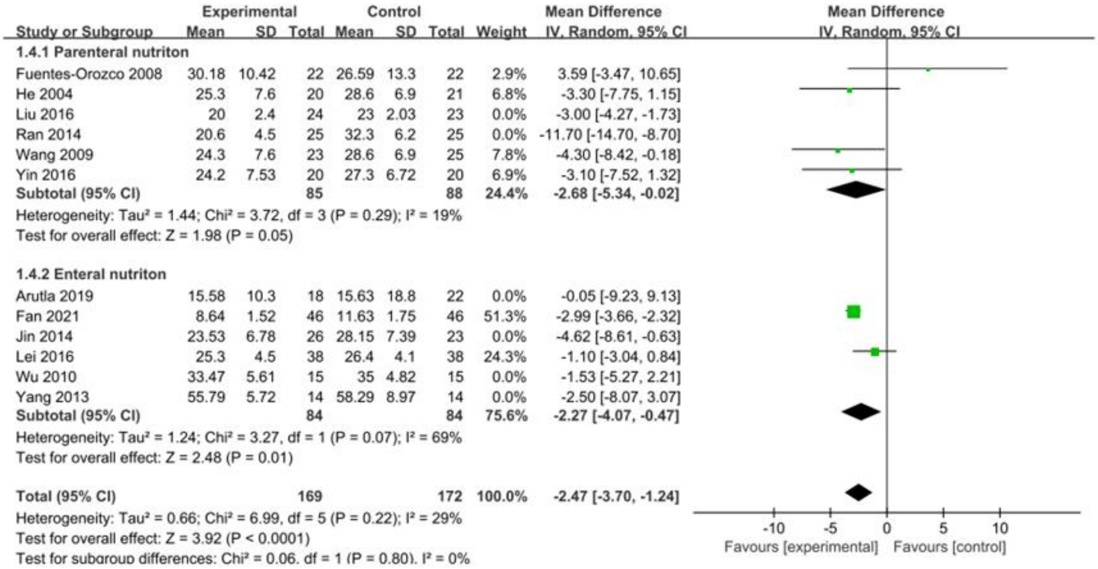

1. Effects of parenteral or enteral nutrition on total hospital stay at 0.4 g/kg Gln supplementation. I^2 tests and *P* are the criteria for the heterogeneity test, ◆: pooled mean difference, —■—: mean difference, and the edges of ◆: 95% CI.

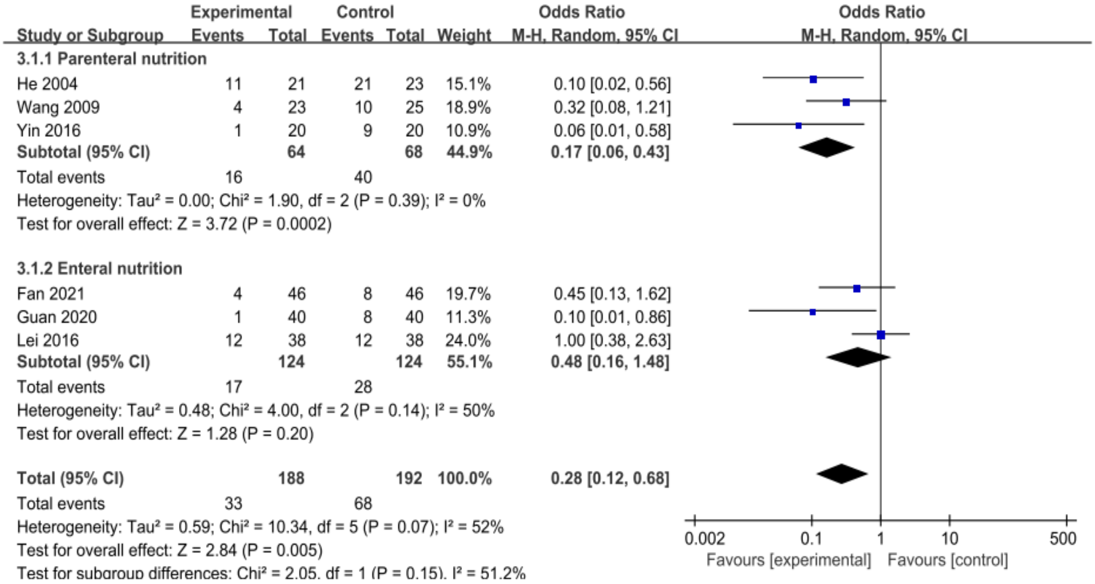

2. Effects of parenteral or enteral nutrition on complications at 0.4 g/kg Gln supplementation. I^2 tests and *P* are the criteria for the heterogeneity test, ◆: pooled odds ratio, —■—: odds ratio, and the edges of ◆: 95% CI.

Study or Subgroup	Experimental Mean	Experimental SD	Experimental Total	Control Mean	Control SD	Control Total	Weight	Std. Mean Difference IV, Random, 95% CI
**3.2.1 Parenteral nutrition**								
Fuentes-Orozco 2008	2.68	0.51	22	2.31	0.62	22	7.3%	0.64 [0.03, 1.25]
Guo 2006	36.8	4.57	20	29.5	4.62	21	6.6%	1.56 [0.85, 2.27]
He 2004	36.8	4.57	20	35.7	4.62	21	7.2%	0.23 [-0.38, 0.85]
Huang 2010	36.6	4.5	24	31.2	4	24	7.2%	1.25 [0.62, 1.87]
Wang 2009	35.8	5.57	23	28.5	5.62	23	7.1%	1.28 [0.64, 1.92]
Yin 2016	37.1	4.47	20	36.1	4.53	20	7.2%	0.22 [-0.40, 0.84]
**Subtotal (95% CI)**			**129**			**131**	**42.7%**	**0.85 [0.40, 1.31]**
Heterogeneity: Tau² = 0.22; Chi² = 15.41, df = 5 (P = 0.009); I² = 68%								
Test for overall effect: Z = 3.67 (P = 0.0002)								
**3.2.2 Enteral nutrition**								
Chu 2020	30.02	2.85	42	25.26	2.77	42	8.0%	1.68 [1.18, 2.18]
Cui 2018	29.36	3.47	47	25.36	2.81	47	8.4%	1.26 [0.81, 1.70]
Fan 2021	36.54	4.65	46	30.54	3.24	46	8.2%	1.48 [1.02, 1.95]
Sun 2019	29.94	2.97	39	25.14	2.73	39	7.9%	1.67 [1.15, 2.18]
Wang 2017	33.45	5.61	49	31.21	4.91	49	8.6%	0.42 [0.02, 0.82]
Yang 2021	34.26	3.25	33	28.67	4.13	32	7.6%	1.49 [0.94, 2.04]
Zhao 2017	33.4	5.6	48	31.2	4.9	48	8.6%	0.41 [0.01, 0.82]
**Subtotal (95% CI)**			**304**			**303**	**57.3%**	**1.19 [0.75, 1.62]**
Heterogeneity: Tau² = 0.29; Chi² = 36.76, df = 6 (P < 0.00001); I² = 84%								
Test for overall effect: Z = 5.36 (P < 0.00001)								
**Total (95% CI)**			**433**			**434**	**100.0%**	**1.04 [0.73, 1.36]**
Heterogeneity: Tau² = 0.26; Chi² = 55.02, df = 12 (P < 0.00001); I² = 78%								
Test for overall effect: Z = 6.51 (P < 0.00001)								
Test for subgroup differences: Chi² = 1.09, df = 1 (P = 0.30), I² = 8.1%								

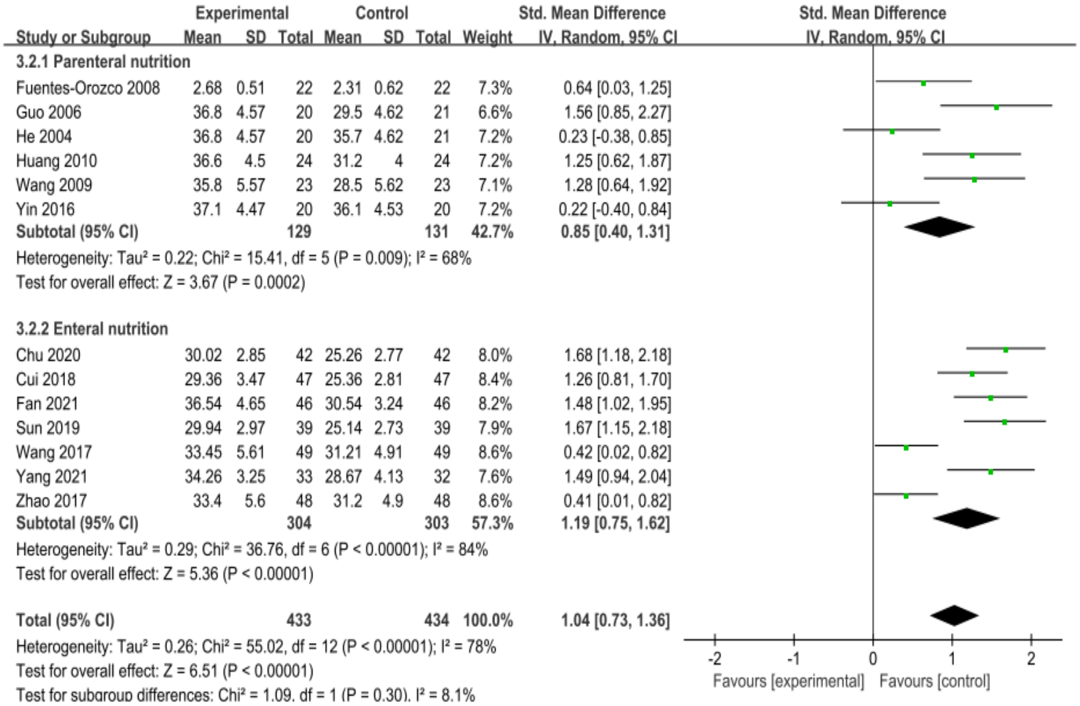

3. Effects of parenteral or enteral nutrition on serum albumin at 0.4 g/kg Gln supplementation. I^2 tests and *P* are the criteria for the heterogeneity test, ◆: pooled standard mean difference, —■—: standard mean difference, and the edges of ◆: 95% CI.
