# Supplementary material for: Efficacy of Glutamine in Treating Severe Acute Pancreatitis: A Systematic Review and Meta-Analysis
Source: Front Nutr. 2022 Jun 14;9:865102. doi: 10.3389/fnut.2022.865102 (PMC9237617; doi:10.3389/fnut.2022.865102)
Supplement: Supplementary file 5 [file Data_Sheet_5.PDF]

Table 1. The original data of mortality associated with experimental group (parenteral or enteral nutrition group supplemented with Gln) vs. control group (conventional nutrition group).

ID	Author and year	TPN+Gln		TPN	
		Events	Total	Events	Total
1	He et al 2004	0	20	3	21
2	Guo et al 2006	0	20	3	21
3	Fuentes-Orozco et al 2008	2	20	5	22
4	Wang et al 2009	0	23	3	25
5	Liu et al 2016	1	23	4	23
6	Yin et al 2016	0	20	3	20

ID	Author and year	EEN+Gln		EEN	
		Events	Total	Events	Total
1	Wu et al 2010	1	14	2	15
2	Jin et al 2014	0	26	0	23
3	Lei et al 2016	2	36	3	38
4	Ren et al 2019	2	28	2	30
5	Fan et al 2021	1	45	4	46
6	Singh et al 2014	5	41	6	39
7	Arutla et al 2019	1	18	1	21

Table 2. The original data of APACHE II score associated with experimental group (parenteral or enteral nutrition group supplemented with Gln) vs. control group (conventional nutrition group).

ID	Author and year	Experimental			Control		
		Mean	SD	Total	Mean	SD	Total
1	Tong et al 2009	7.57	2.16	20	7.65	2.7	20
2	Yang et al 2013	5.93	0.92	14	5.79	0.69	14
3	Jin et al 2014	3.1	1.3	26	5.4	1.4	23
4	Wa et al 2015	4.7	0.5	54	5.4	0.6	54
5	Lei et al 2016	13.1	3.5	38	14.8	3.5	38
6	Zhao et al 2017	4.7	0.5	48	5.3	0.6	48
7	Gao et al 2018	4.67	0.53	45	5.54	0.74	45
8	Ren et al 2019	4.03	0.76	30	5.34	1.04	30
9	Fan et al 2021	3.98	0.62	46	5.62	0.76	46
10	Yang et al 2021	7.03	1.42	33	8.72	1.38	32
11	Huang et al 2010	10.2	3.4	24	15.2	3	24
12	Ran et al 2014	4.3	1.8	25	4.6	1.6	25

Table 3. The original data of ICU hospital stay associated with experimental group (parenteral or enteral nutrition group supplemented with Gln) vs. control group (conventional nutrition group).

ID	Author and year	Experimental			Control		
		Mean	SD	Total	Mean	SD	Total
1	Fuentes-Orozco et al 2008	11	11.7	22	11.14	7.41	22
2	Ran et al 2014	20.6	4.5	25	32.3	6.2	25
3	Liu et al 2016	11.5	2	24	15.2	2	23
4	Yang et al 2013	2.57	0.85	14	3.64	1.6	14
5	Arutla et al 2019	8.31	5.8	18	7.9	11.7	22

Table 4. The original data of total length of hospital stay associated with experimental group (parenteral or enteral nutrition group supplemented with Gln) vs. control group (conventional nutrition group).

ID	Author and year	TPN+Gln			TPN		
		Mean	SD	Total	Mean	SD	Total
1	He et al 2004	25.3	7.6	20	28.6	6.9	21
2	Fuentes-Orozco et al 2008	30.18	10.42	22	26.59	13.3	22
3	Wang et al 2009	24.3	7.6	23	28.6	6.9	25
4	Ran et al 2014	20.6	4.5	25	32.3	6.2	25
5	Liu et al 2016	20	2.4	24	23	2.03	23
6	Yin et al 2016	24.2	7.53	20	27.3	6.72	20

ID	Author and year	EEN+Gln			EEN		
		Mean	SD	Total	Mean	SD	Total
1	Wu et al 2010	33.47	5.61	15	35	4.82	15
2	Yang et al 2013	55.79	5.72	14	58.29	8.97	14
3	Jin et al 2014	23.53	6.78	26	28.15	7.39	23
4	Lei et al 2016	25.3	4.5	38	26.4	4.1	38
5	Fan et al 2021	8.64	1.52	11.63	1.75	46	23
6	Arutla et al 2019	15.58	10.3	15.63	18.8	22	20

Table 5. The original data of bloating recovery time associated with experimental group (parenteral or enteral nutrition group supplemented with Gln) vs. control group (conventional nutrition group).

ID	Author and year	Experimental			Control		
		Mean	SD	Total	Mean	SD	Total
1	He et al 2004	5.6	0.87	20	7.3	1.78	21
2	Wang et al 2009	6.4	2.09	23	7.3	1.78	25
3	Yin et al 2016	6.1	1.22	20	7.8	2.31	20
4	Ren et al 2019	2.28	0.33	30	3.37	0.42	30
5	Fan et al 2021	5.87	1.29	46	8.42	1.35	46

Table 6. The original data of complications associated with experimental group (parenteral or enteral nutrition group supplemented with Gln) vs. control group (conventional nutrition group).

ID	Author and year	TPN+Gln		TPN	
		Events	Total	Events	Total
1	He et al 2004	11	21	21	23
2	Wang et al 2009	4	23	10	25
3	Liu et al 2016	6	24	11	23
4	Yin et al 2016	1	20	9	20

ID	Author and year	EEN+Gln		EEN	
		Events	Total	Events	Total
1	Wu et al 2010	3	15	5	15
2	Jin et al 2014	9	26	11	23
3	Lei et al 2016	12	38	12	38
4	Ren et al 2019	2	30	5	30
5	Guan et al 2020	1	40	8	40
6	Fan et al 2021	4	46	8	46
7	Singh et al 2014	21	41	19	239

Table 7. The original data of serum albumin associated with experimental group (parenteral or enteral nutrition group supplemented with Gln) vs. control group (conventional nutrition group).

ID	Author and year	TPN+Gln			TPN		
		Mean	SD	Total	Mean	SD	Total
1	He et al 2004	36.8	4.57	20	35.7	4.62	21
2	Guo et al 2006	36.8	4.57	20	29.5	4.62	21
3	Fuentes-Orozco et al 2008	2.68	0.51	22	2.31	0.62	22
4	Gu et al 2008	37.8	7.5	10	28.3	6.7	10
5	Wang et al 2009	35.8	5.57	23	28.5	5.62	23
6	Huang et al 2010	36.6	4.5	24	31.2	4	24
7	Yin et al 2016	37.1	4.47	20	36.1	4.53	20

ID	Author and year	EEN+Gln			EEN		
		Mean	SD	Total	Mean	SD	Total
1	Wu et al 2010	40.01	2.71	15	38.5	2.51	15
2	Wang et al 2017	33.45	5.61	49	31.21	4.91	49
3	Zhao et al 2017	33.4	5.6	48	31.2	4.9	48
4	Cui et al 2018	29.36	3.47	47	25.36	2.81	47
5	Sun et al 2019	29.94	2.97	39	25.14	2.73	39
6	Chu et al 2020	30.02	2.85	42	25.26	2.77	42
7	Fan et al 2021	36.54	4.65	46	30.54	3.24	46
8	Yang et al 2021	34.26	3.25	33	28.67	4.13	32

Table 8. The original data of ALT associated with experimental group (parenteral or enteral nutrition group supplemented with Gln) vs. control group (conventional nutrition group).

ID	Author and year	Experimental			Control		
		Mean	SD	Total	Mean	SD	Total
1	Wa et al 2015	24.6	2.1	54	34.3	2.8	54
2	Wang et al 2017	23.65	2.23	49	35.13	2.71	49
3	Zhao et al 2017	23.6	2.2	48	35.1	2.7	48
4	Gao et al 2018	23.42	2.26	45	35.39	2.74	45
5	Sun et al 2019	30.68	4.14	39	49.68	3.57	39
6	Chu et al 2020	30.83	4.06	42	50.02	3.27	42

Table 9. The original data of AST associated with experimental group (parenteral or enteral nutrition group supplemented with Gln) vs. control group (conventional nutrition group).

ID	Author and year	Experimental			Control		
		Mean	SD	Total	Mean	SD	Total
1	Wa et al 2015	22.1	3.2	54	31.3	3.7	54
2	Sun et al 2019	21.96	4.87	39	49.75	3.96	39
3	Chu et al 2020	22.44	4.82	42	49.85	4.02	42

Table 10. The original data of TBIL associated with experimental group (parenteral or enteral nutrition group supplemented with Gln) vs. control group (conventional nutrition group).

ID	Author and year	Experimental			Control		
		Mean	SD	Total	Mean	SD	Total
1	Wa et al 2015	11.2	1.4	54	16.1	1.7	54
2	Wang et al 2017	12.31	4.21	49	17.32	1.51	49
3	Zhao et al 2017	12.3	1.1	48	17.3	1.5	48
4	Gao et al 2018	12.27	1.1	45	17.44	1.58	45

Table 11. The original data of Scr associated with experimental group (parenteral or enteral nutrition group supplemented with Gln) vs. control group (conventional nutrition group).

ID	Author and year	Experimental			Control		
		Mean	SD	Total	Mean	SD	Total
1	Ran et al 2014	79.6	60.2	25	101.2	58.6	25
2	Wa et al 2015	109.1	9.3	54	112.3	11.5	54
3	Sun et al 2019	97.38	14.02	39	114.16	14.18	39
4	Chu et al 2020	97.56	14.12	42	114.23	14.08	42

Table 12. The original data of BUN associated with experimental group (parenteral or enteral nutrition group supplemented with Gln) vs. control group (conventional nutrition group).

ID	Author and year	Experimental			Control		
		Mean	SD	Total	Mean	SD	Total
1	Ran et al 2014	3.7	6.5	25	4.3	5.8	25
2	Wa et al 2015	5.2	1.4	54	5.1	1.4	54
3	Sun et al 2019	3.38	1.57	39	5.61	1.7	39
4	Chu et al 2020	3.43	1.61	42	5.68	1.73	42

Table 13. The original data of CRP associated with experimental group (parenteral or enteral nutrition group supplemented with Gln) vs. control group (conventional nutrition group).

ID	Author and year	TPN+Gln			TPN		
		Mean	SD	Total	Mean	SD	Total
1	Fuentes-Orozco et al 2008	52.04	31.9	22	95.27	60.9	22
2	Huang et al 2010	15.4	3.5	24	21	4	24
3	Ran et al 2014	30.2	6.3	25	39.8	6.5	25

ID	Author and year	EEN+Gln			EEN		
		Mean	SD	Total	Mean	SD	Total
1	Tong et al 2009	60.3	27.8	20	83.3	33.4	20
2	Yang et al 2013	54.36	3.67	14	57.93	5.54	14
3	Yuan et al 2018	56.31	18.04	23	98.12	11.54	24
4	Ren et al 2019	3.86	0.62	30	7.72	1.25	30

Table 14. The original data of IL-6 associated with experimental group (parenteral or enteral nutrition group supplemented with Gln) vs. control group (conventional nutrition group).

ID	Author and year	Experimental			Control		
		Mean	SD	Total	Mean	SD	Total
1	Tong et al 2009	107.5	8.9	20	114.7	22.6	20
2	Wa et al 2015	4.9	1.1	54	5.7	1.5	54
3	Lei et al 2016	14.79	5.23	38	17.87	6.06	38
4	Wang et al 2017	4.82	1.23	49	5.91	1.31	49
5	Yang et al 2017	23.41	3.46	34	35.31	4.67	34
6	Zhao et al 2017	4.8	1.2	48	5.9	1.3	48
7	Cui et al 2018	4.33	1.24	47	9.35	2.28	47
8	Gao et al 2018	4.77	1.14	45	5.99	1.36	45
9	Yuan et al 2018	45.87	10.99	23	54.72	4.77	24
10	Ren et al 2019	8.52	1.36	30	13.37	2.02	30
11	Guan et al 2020	4.46	1.35	40	5.89	1.23	40

Table 15. The original data of IL-8 associated with experimental group (parenteral or enteral nutrition group supplemented with Gln) vs. control group (conventional nutrition group).

ID	Author and year	Experimental			Control		
		Mean	SD	Total	Mean	SD	Total
1	Wa et al 2015	10.6	1.8	54	11.9	2.1	54
2	Wang et al 2017	10.23	2.158	49	16.21	2.73	49
3	Cui et al 2018	11.54	2.47	47	18.47	2.56	47
4	Yuan et al 2018	45.15	9.27	23	59.03	4.87	24
5	Guan et al 2020	9.89	2.16	40	15.89	2.45	40

Table 16. The original data of TNF-α associated with experimental group (parenteral or enteral nutrition group supplemented with Gln) vs. control group (conventional nutrition group).

ID	Author and year	Experimental			Control		
		Mean	SD	Total	Mean	SD	Total
1	Wa et al 2015	7.2	1.5	54	10.7	1.9	54
2	Lei et al 2016	25.04	3.43	38	24.48	3.15	38
3	Wang et al 2017	7.13	1.41	49	12.21	1.81	49
4	Yang et al 2017	67.53	9.34	34	99.52	11.25	34
5	Zhao et al 2017	7.1	1.4	48	12.2	1.8	48
6	Cui et al 2018	7.48	2.13	47	19.38	5.19	47
7	Gao et al 2018	7.06	1.44	45	12.19	1.79	45
8	Yuan et al 2018	24.35	7.15	23	32.41	4.22	24
9	Ren et al 2019	8.31	1.25	30	15.71	3.7	30
10	Guan et al 2020	7.23	1.54	40	13.53	2.01	40

Table 17. The original data of IgA associated with experimental group (parenteral or enteral nutrition group supplemented with Gln) vs. control group (conventional nutrition group).

ID	Author and year	Experimental			Control		
		Mean	SD	Total	Mean	SD	Total
1	Ding et al 2007	1679	149.6	10	1596	429.5	10
2	Fuentes-Orozco et al 2008	407.27	114.33	22	321.41	159	22
3	Ran et al 2014	2.4	0.2	25	1.7	0.2	25

Table 18. The original data of IgG associated with experimental group (parenteral or enteral nutrition group supplemented with Gln) vs. control group (conventional nutrition group).

ID	Author and year	Experimental			Control		
		Mean	SD	Total	Mean	SD	Total
1	Ding et al 2007	8.59	0.46	10	7.33	0.84	10
2	Huang et al 2010	19.6	4	24	16	5.2	24
3	Ran et al 2014	9.8	1.2	25	8.7	1	25

Table 19. The original data of serum amylase recovery time associated with experimental group (parenteral or enteral nutrition group supplemented with Gln) vs. control group (conventional nutrition group).

ID	Author and year	Experimental			Control		
		Mean	SD	Total	Mean	SD	Total
1	He et al 2004	6	1.21	20	5.6	0.87	21
2	Wang et al 2009	6	1.21	23	5.6	0.87	25
3	Yin et al 2016	6.2	2.08	20	5.2	0.91	20
4	Ren et al 2019	4.36	1.12	30	5.28	1.35	30

Table 20. The original data of response rate associated with experimental group (parenteral or enteral nutrition group supplemented with Gln) vs. control group (conventional nutrition group).

ID	Author and year	Experimental			Control		
		Mean	SD	Total	Mean	SD	Total
1	Lei et al 2016	34	4	38	27	11	38
2	Chu et al 2020	40	2	42	33	9	42
3	Yang et al 2021	31	2	33	23	9	32
